# Induction of circulating T follicular helper cells and regulatory T cells correlating with HIV-1 gp120 variable loop antibodies by a subtype C prophylactic vaccine tested in a Phase I trial in India

**DOI:** 10.1371/journal.pone.0203037

**Published:** 2018-08-29

**Authors:** Sivasankaran Munusamy Ponnan, Soumya Swaminathan, Kannan Tiruvengadam, Vidyavijayan K. K., Narayana Cheedarla, Manohar Nesakumar, Sujitha Kathirvel, Rajat Goyal, Nikhil Singla, Joyeeta Mukherjee, Philip Bergin, Jakub T. Kopycinski, Jill Gilmour, Srikanth Prasad Tripathy, Hanna Elizabeth Luke

**Affiliations:** 1 National Institute for Research in Tuberculosis (Indian Council of Medical Research), Chennai, India; 2 International AIDS Vaccine Initiative, New Delhi, India; 3 IAVI Human Immunology Laboratory, Imperial College, London, United Kingdom; George Washington University School of Medicine and Health Sciences, UNITED STATES

## Abstract

A Phase I HIV-1 vaccine trial sponsored by the International AIDS Vaccine Initiative (IAVI) was conducted in India in 2009 to test a subtype C prophylactic vaccine in a prime-boost regimen comprising of a DNA prime (ADVAX) and MVA (TBC-M4) boost. The trial demonstrated that the regimen was safe and well tolerated and resulted in enhancement of HIV-specific immune responses. Preliminary observations on vaccine-induced immune responses were limited to analysis of neutralizing antibodies and IFN-γ ELISPOT response. The present study involves a more detailed analysis of the nature of the vaccine-induced humoral immune response using specimens that were archived from the volunteers at the time of the trial. Interestingly, we found vaccine induced production of V1/V2 and V3 region-specific antibodies in a significant proportion of vaccinees. Variable region antibody levels correlated directly with the frequency of circulating T follicular helper cells (Tfh) and regulatory T cells (Treg). Our findings provide encouraging evidence to demonstrate the immunogenicity of the tested vaccine. Better insights into vaccine-induced immune responses can aid in informing future design of a successfulHIV-1 vaccine.

## Introduction

According to the recent UNAIDS report, there are 36.7 million people living with HIV worldwide. India alone has 2.1 million people living with HIV and has reported approximately 68,000 deaths due to AIDS-related illnesses [1]. The increasing burden of HIV presents the urgent need for a vaccine to curb the pandemic. Although several vaccine candidates have been tested in various clinical trials, we are still not close to a successful HIV vaccine [2]. The RV144 trial conducted by the Thai government and the US Military has been the most promising thus far [3].This trial employed a prime-boost vaccination regimen comprising of a non-replicating recombinant canary pox vector ALVAC-HIV (vCP1521) prime and AIDSVAX gp120 B/E boost, and demonstrated that induction of antibodies to the V1/V2 peptides of the HIV-1 envelope correlated with a lower risk of infection, thus becoming the first large-scale Phase III HIV vaccine trial to exhibit a modest level of protective efficacy [4,5].

In 2009, the National Institute for Research in Tuberculosis (formerly Tuberculosis Research Centre) at Chennai, India, and the National AIDS Research Institute at Pune, India, undertook an IAVI-sponsored Phase I HIV-1 subtype C prophylactic vaccine trial, known as the P001 trial (Clinical Trial registry CTRI/2009/091/000051) [6]. This randomized, placebo controlled, double blind, phase I trial enrolled 16 HIV-uninfected, healthy male and female adult participants at each of the 2 sites. The trial tested the safety and immunogenicity of a heterologous prime-boost regimen employing ADVAX, a DNA-based vaccine consisting of Chinese HIV-1 subtype C env gp160, gag, pol and nef/tat genes cloned into the pVAX1 mammalian expression vector (Lot # 04030248, Vical, Inc., San Diego, CA) as the prime, and TBC-M4 a recombinant (MVA) vector encoding Indian HIV-1 subtype C env gp160, gag, RT, rev, tat, and nef genes (Lot # 1B, Therion Biologics Corporation, Cambridge MA) as the boost, with that of homologous MVA alone. Preliminary investigations found that 3 months after the final booster dose, all volunteers in both the groups had positive HIV-specific antibody responses against the Env, Gag, and Pol proteins. The study also characterized the neutralization ability of the antibodies and demonstrated the presence of neutralizing antibodies capable of neutralizing Tier-1 but not Tier-s pseudo viruses at 14 days post final vaccination. However, the titers decreased at 3 months post last vaccination. Based on these observations it was concluded that the vaccine did not induce production of potent broadly neutralizing antibodies [6].

Most HIV-infected subjects produce neutralizing antibodies (NAbs) in low titers with limited neutralizing activity [7,8].Though NAbs tend to protect against infection, constant mutations in the virus helps them to evade neutralization by these antibodies [9]. A very small proportion of HIV-infected individuals produce Nabs that can cross-neutralize a large number of viral strains. These antibodies, called bNAbs, have been isolated from individuals referred to as elite neutralizers [10, 11].Non-neutralizing antibodies (n-NAbs) also possess antiviral property, but their role in preventing infection is still naïve. HIV-1 specific Fc-gamma receptors of vaccine-induced antibodies activate mediators of antibody dependent cellular cytotoxicity (ADCC). Studies have shown that elite responders develop stronger and broader ADCC or neutralizing responses that are also potent against HIV-1 mutants.

Recent studies have reported that elite controller-derived cells interact strongly with B cells promoting their maturation, and class switch recombination [12].Tfh cells are a subset of CD4+T cells defined by CXCR5^hi^ PD-1^hi^ expression that reside in the lymphoid follicles [13, 14] and provide help to B cells for affinity maturation and differential response to pathogens [15]. Interaction of Tfh cells with high-affinity germinal center B cells is crucial for B cell survival, affinity maturation, and antibody class switching. Interleukin-21 (IL-21) secreted by Tfh cells is required for the generation of memory B cells and plasma cells (PCs). IL-21 synergistically acts with CD40L to activate B cells [16, 17]. High frequency of functional circulating PD-1^+^CXCR3^−^CXCR5^+^ memory peripheral Tfh (pTfh) cells have been reported to correlate with high titres of HIV-specific broadly neutralizing antibodies (bNAbs) and reduced viral loads in HIV-infected individuals [18]. Interestingly, the RV144 vaccine, which induced production of V1/V2 antibodies that correlated with protection against HIV infection, also induced IL-21 secreting pTfh cells in the vaccinees [19]. Thus, measuring vaccine-induced pTfh and B cell responses may provide key insights into the ability of the vaccine to elicit antibody-mediated protective immune responses.

Regulatory T (Treg) cells down regulate the hyperactive immune system and act as immuno suppressors during HIV infection. Besides, they also regulate peripheral tolerance. Tregs can interact with microbial pathogens to sustain the delicate balance between the host and the pathogen [20–22].Studies have documented that increased frequency of Tregs dampen HIV-specific immune responses [23].The naïve and effector Treg subsets possess unique functions in auto-immunity, immune surveillance, and disease progression [24–25]. However, the functional heterogeneity of Treg subsets and their involvement in immune-regulation during HIV vaccination remains largely unexplored and can provide valuable insights to improve vaccine immunogenicity.

The present study describes a more detailed characterization of the humoral immune responses induced by the P001 vaccine.

## Materials and methods

### Ethics statement

The P001 vaccine trial was conducted with the approval of the Institutional Ethics Committee of NIRT and samples were collected and stored appropriately with the written informed consent of the volunteers. Samples archived during the conduct of the trial for future studies were used for the present study with the approval of the NIRT IEC [NIRT IEC N0-2015013].

### Study samples

**Fig 1:**
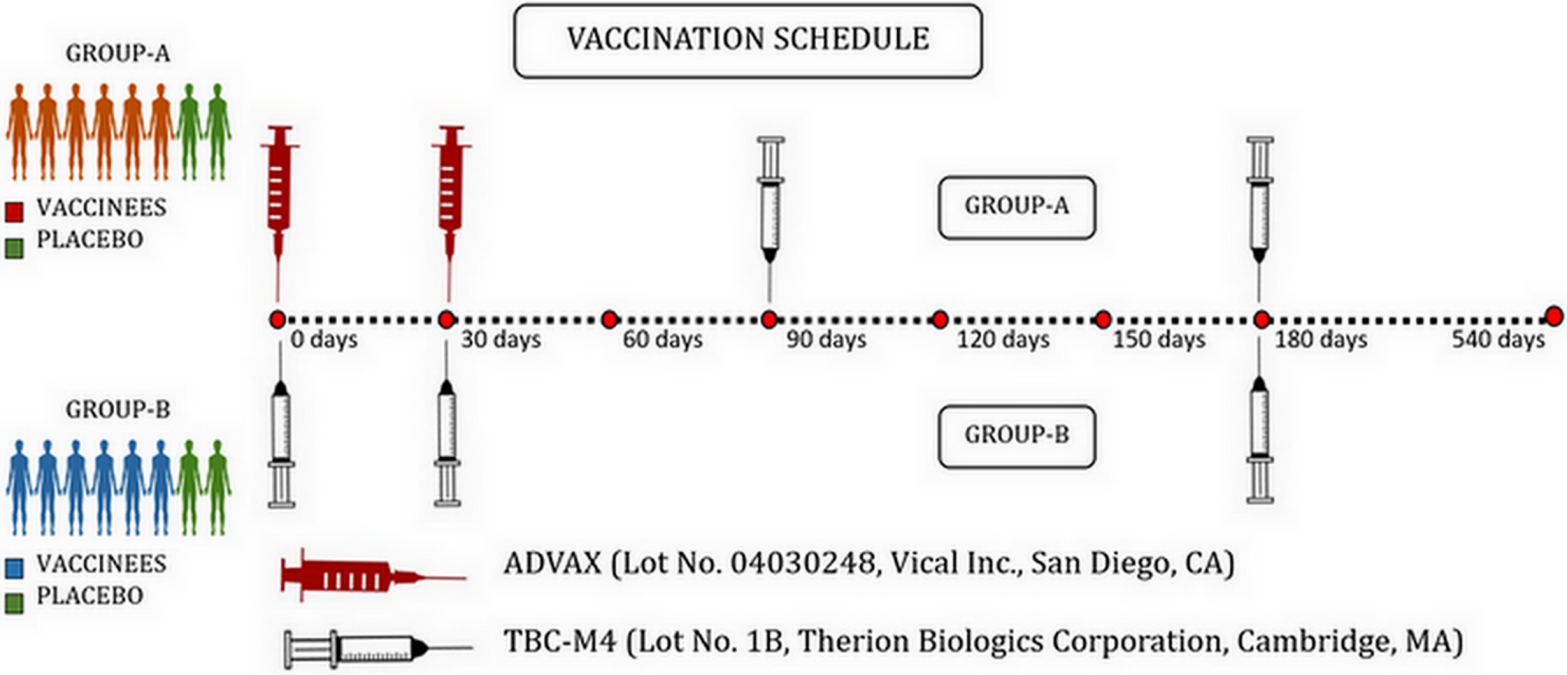
Vaccination schedule of IAVI Phase-I prime boost HIV-1 Subtype-C prophylactic vaccine Trial-NIRT-ICMR (P001 Trial).

Plasma and cryopreserved peripheral blood mononuclear cells (PBMCs) archived at NIRT alone were accessible and used for the present study. The samples belonged to 16 volunteers (9 males and 7 females) who were randomly assigned to either group A or B, with eight participants in each group. Group A participants received two intramuscular (I.M.) injections of ADVAX (a DNA vaccine), or placebo at baseline (time ‘0’) and 1 month, followed by two I.M. injections of TBC-M4 (a recombinant MVA), or placebo at months 3 and 6. Group B participants received three I.M. injections of TBC-M4 or placebo at time 0, month 1 and month 6. Placebo used was 1X sterile PBS with 10% glycerol. Among the 8 volunteers in each group, 6 received the vaccine and 2 received placebo. Fig 1 depicts the schema of the vaccination strategy.

### Analysis of anti-V1, V2, V3 and MPER-specific antibodies

Plasma samples collected at baseline as well as at 2 weeks after the first and last vaccination time point were tested for the presence of IgG antibodies specific to V1, V2, V3 and MPER peptides of HIV-1 envelope gp120 using direct ELISA. Briefly, 96 well plates were coated with 5 μg/ml of peptide pools (15-mer peptides with 11 amino acid overlap) constituting the V1, V2, V3 and MPER regions, in 100 mM NaHCO_3 (_pH 9.6), by overnight incubation at 4°C. Plates were washed with PBST (PBS containing 0.05% Tween 20) and blocked with PBS (Lonza, India) containing 1% BSA and 0.05% Tween 20 at 37°C for 2 hours. After washing, heat-inactivated plasma samples diluted 1:50 with diluent (ABL Inc., MD) were added to each well and incubated at 37°C for 1 hour. Subsequently, plates were washed and incubated with HRP-conjugated goat anti-human IgG (Thermo Fisher, Waltham, MA) at a dilution of 1:120000 at 37°C for 1 hour. Plates were developed using One-step TMB substrate (Thermo Fisher). The reaction was stopped using 1N H_2_SO_4_ and plates were read at 450nm using a microplate reader (ELx808-BioTek). All tests were performed in duplicates. Pooled normal healthy plasma was used as the negative control. The cut-off for a positive response was defined as mean OD of the negative samples plus 3 times the standard deviation (Table 1).

**Table 1:** Variable region and MPER peptides used to study the reactivity of plasma specimens from the vaccinees and placebo recipients. (15-mer peptides with 11 amino acid overlap).

**V1**	CRNVSLCVTLECRNVSSNGTLECRNVSSNGTYNETLECRNVSSNGTYNETRNVSSNGTYNETYNEISNGTYNETYNEIKNCSYNETYNEIKNC
**V2**	SFNATYNEIKNCSFNATTVLRKNCSFNATTVLRDRKQFNATTVLRDRKQTVYAVLRDRKQTVYALFYRRKQTVYALFYRLDIVVYALFYRLDIVPLNKFYRLDIVPLNKKNSSDIVPLNKKNSSENSSLNKKNSSENSSEYYRKNSSENSSEYYRLINC
**V3**	CTRPNNNTRIVCTRPNNNTRKSIRRPNNNTRKSIRIGPGTRKSIRIGPGQTFYSIRIGPGQTFYATGDGPGQTFYATGDIIGDTFYATGDIIGDIRQATGDIIGDIRQAHC
**MPER**	LDERNEKDLLALDNWKNKDLLALDNWKNLWSWALDNWKNLWSWFDITWKNLWSWFDITNWLWWSWFDITNWLWYIRIDITNWLWYIR

### Evaluation of vaccine-induced T and B cells subsets by multicolour immunophenotyping

Cryopreserved PBMC that were collected one week after second vaccination and after 1, 2 and 48 weeks after last vaccination were used for this analysis. Cells were thawed, washed with 10% CRPMI and viable cell count was determined using the trypan blue dye exclusion method. cell viability was >90% and recovery was >70%. The cells cells were rested overnight, stained with an amine-reactive viability dye (Live/Dead aqua, Life Technologies) for 20 minutes at room temperature (Chowdhury et al 2015) [26], washed with FACS buffer and stained with the following cocktail of monoclonal antibodies: T follicular helper cell (Tfh) panel: CD3-APC H7, CD4-BUV737, CD45RO-BUV395, CCR7-PEcy7, CXCR3-APC Alexa 700, CXCR5-BB515, and PD-1-PE; B cell panel: CD3-APC H7, CD38-APC, CD20-PE, CD19-BUV395, IgD-BUV737, CD27- BB515; regulatory T cell (Treg) panel: CD3-APC H7, CD4-BUV737, CD45RO-BUV395, CCR7-PEcy7, CD25-APC and CD127-PECF 594, for 20 minutes at 4 ºC (antibody and clone description are provided in S1 Table). About 2 × 10^6^ cells were stained for each panel. After staining, the cells were washed, fixed with BD Cytofix (2% paraformaldehyde) and analyzed on a FACS ARIA III flow cytometer (Becton Dickinson). A minimum of 1,000,000 total events were acquired and data were analyzed using FlowJo software, version 10 (Tree star Inc., Ashland, Oregon, USA).

### Statistical analysis

Statistical analyses were performed using GraphPad Prism, version 5 (GraphPad Software, Inc., CA). Values are presented as median, interquartile, and percentage. Two-way ANOVA was used to examine the difference in frequency (%) of different immune cell subsets during the vaccination period. Bonferroni post hoc test was used for sub-group analysis. Correlation analysis was performed to determine the relationship between frequency of different immune cell types and variable region specific antibody responses (OD values). For all analyses, differences were considered significant if p value was <0.05.

## Results

### Induction of HIV-1 gp120 variable loop specific antibody responses

**Fig 2:**
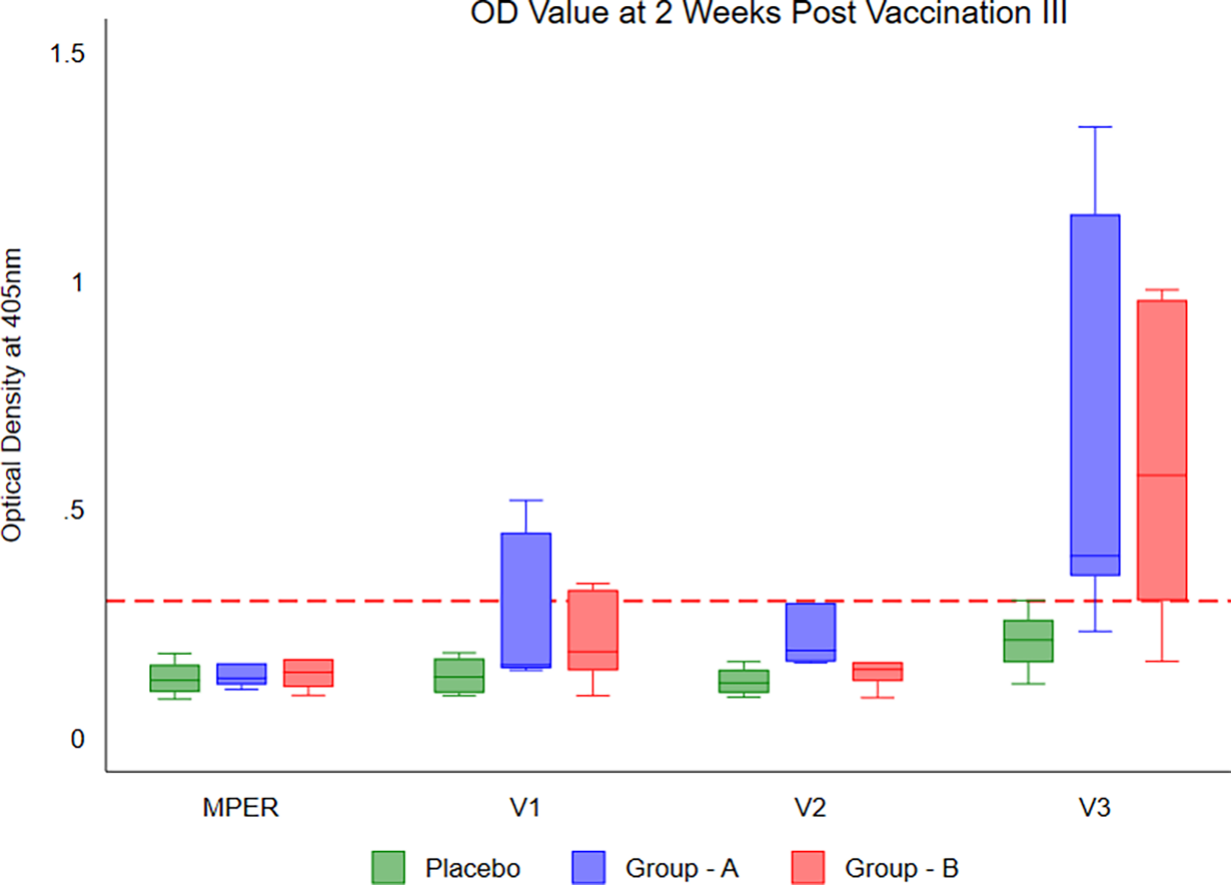
Reactivity of plasma obtained volunteers at two weeks after last vaccination to the variable loop and MPER peptides. (Note: 2wk+Post VAC-3 –two weeks after the last MVA booster dose).Values represent ELISA-generated optical density (OD) values at wavelength 405nM. Plasma was tested at a dilution of 1:50. Two-way ANOVA using Bonferroni post-hoc test was used at 5%* level of significance.

Plasma samples obtained prior to vaccination as well as at 2 weeks post first and last MVA vaccination were analyzed for IgG antibodies specific to the first three variable loops (V1, V2 and V3) of the HIV-1 envelope as well as the MPER region. While none of the samples had binding antibodies at baseline or immediately after first vaccination, variable loop antibodies were detected after MVA vaccination (Fig 2). The antibody levels increased consistently with each booster dose of MVA and reached peak values immediately after the last booster (S2 Table). Antibodies to the V1 peptide were detected among 40% (2/6) of Group A and 50% (3/6) of Group B volunteers. Antibodies to V2 peptide were detected among 50% (3/6) of Group A and 40% (2/6) of Group B volunteers. V3-specific antibodies were detected in 90% (5/6) of Group A and Group B volunteers. On the other hand, MPER-binding antibodies were detected only in 10% (1/6) of Group A and Group B volunteers. These findings reveal that the poxvirus and adenovirus based HIV vaccines were capable of inducing antibodies with variable regions binding specificity similar to that seen in the previous HIV-1 vaccine trials [27, 28].

### Induction of circulating T follicular helper cells correlating with levels of variable loop antibodies

PBMC from the pre-vaccination, one week post second vaccination and 1, 2 and 48 weeks post final vaccination time points were analyzed by multicolor flow cytometry to enumerate the number of pTfh cells (S1 Table) defined as CD4+CD45RO+CCR7+PD1+CXCR5+CXCR3- cells as described by Locci et al [18] (S1 Fig).

**Fig 3:**
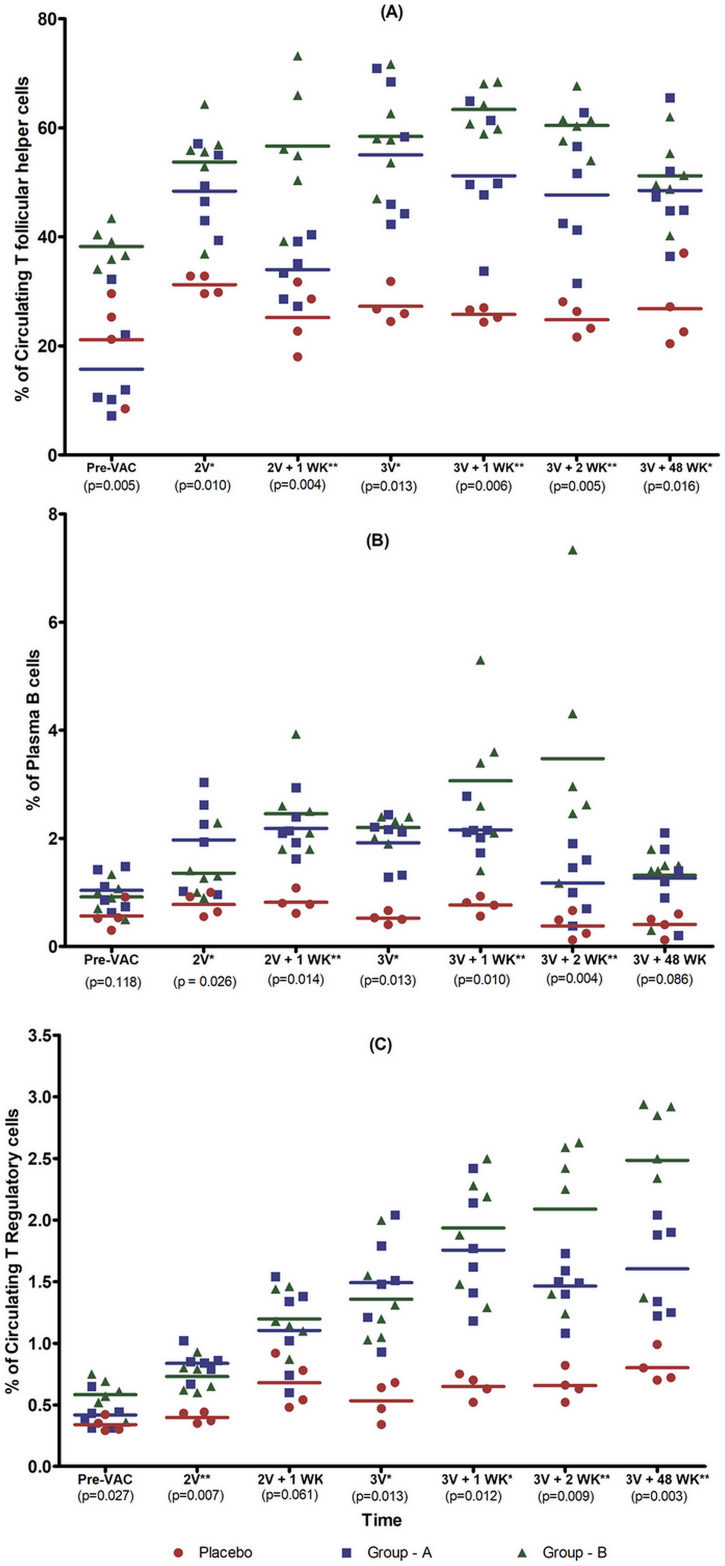
Vaccine induced immune cell subsets in PBMC samples (Note: 2V - First MVA, 2V+1wk—one week after first MVA vaccination, 3V - Last MVA booster dose, 3V+1wk—one week after final MVA booster dose, 3V+2wk—two weeks after final MVA dose, 3V+48wk—48 weeks after final MVA booster). **(3A)** Frequency of vaccine induced circulating T follicular helper cells in Groups A and B after 2^nd^ vaccination, 1-week post 2^nd^ vaccination, 3^rd^ vaccination, and 1^st^ week, 2^nd^ week, and 48^th^ week post 3^rd^ vaccination. **(3B)** Frequency of Plasma B cells in Groups A and B after 2^nd^ vaccination, 1^st^ week post 2^nd^ vaccination, 3^rd^ vaccination, 1^st^ week, 2^nd^ week and 48 weeks post 3^rd^ vaccination **(3C)** Frequency of Circulating Regulatory cells T cells in Groups A and B after 2^nd^ vaccination, 1^st^ week post 2^nd^ vaccination, 3^rd^ vaccination, 1^st^ week, 2^nd^ week and 48 weeks post 3^rd^ vaccination. Graphical representation of the frequency of immune cells in placebo and vaccinees of both groups at different time points. Horizontal bars represent mean values and P values were calculated using two-way ANOVA using Bonferroni post hoc test.

**Fig 4:**
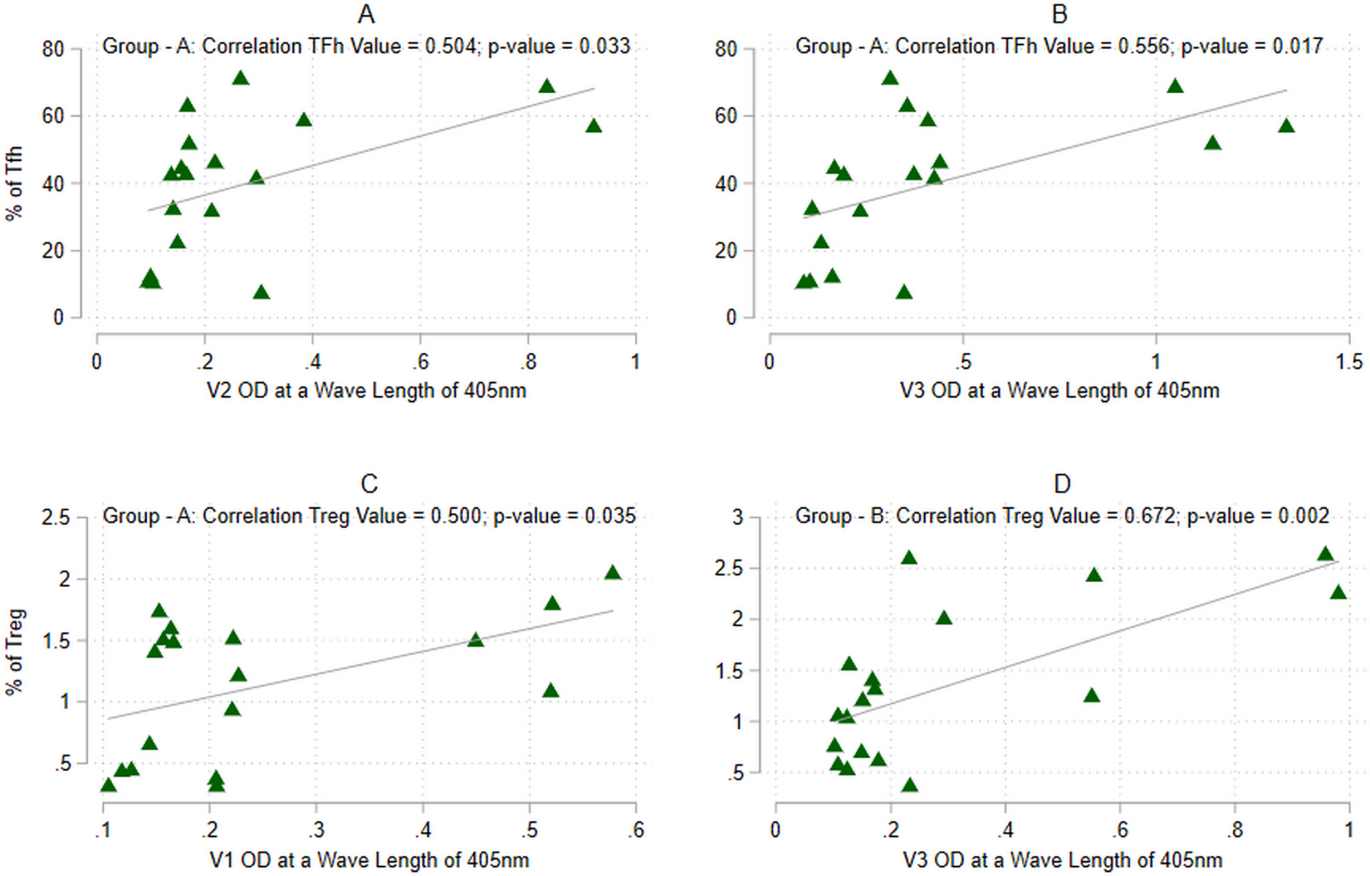
Correlation between immune cells and variable loop and MPER-specific antibodies. (A) Frequency of vaccine induced circulating T follicular helper cells vs antibodies to the V1 antigen. (B) Frequency of vaccine induced circulating T follicular helper cells vs antibodies to the V3 antigen. (C) Frequency of vaccine induced circulating T regulatory cells vs antibodies to the V2 antigen. (D) Frequency of vaccine induced circulating T regulatory cells vs antibodies to the V3 antigen.

Vaccination induced significantly more Tfh cells than did the placebo (Fig 3A). The median frequency of Tfh cells among CD4^+^ T cells was 0.13% (range: 0.061–0.17%) in Group A, 0.2% (range: 0.72–0.2%) in Group B and 0.08% (range: 0.04–0.15%) in the placebo (S3 Table). The numbers of Tfh cells were observed to increase progressively with time in both groups A and B, and remained significantly higher in the vaccine recipients as compared to the placebo (p<0.01). The frequency of circulating memory-like Tfh cells in Group B was significantly higher than in Group A at all the time points (p<0.01; S3 Table). Interestingly, a positive correlation was observed between the V2 and V3 specific antibody response and the number of circulating Tfh cells (p<0.033 and p<0.017 respectively) in Group A alone (Fig 4).

### Vaccine-induced plasma B cells

Plasma cells were identified as CD19+ CD38+ CD27+ cells, memory cells were distinguished based on expression of IgD and CD27 as described by Kaminski et al. [29], class switched memory cells were identified as IgD- CD27+ cells, IgM memory cells as IgD+ CD27+ cells, double negative memory B cells as IgD- CD27- cells, and mature B cells as IgD+ CD27- cells (S2 Fig). At the pre-vaccination time point, all volunteers had comparable numbers of circulating plasma B cells (Fig 3B). Vaccination induced a significant increase in the number of Plasma B cells particularly in group B (p<0.01). The median frequency of Plasma cells was 1.7% (range: 1.11–2.14%) in Group A, 2.0% (range: 1.32–3.01%) in Group B and 0.5% (range: 0.49–0.79%) in the placebo. At the final time point analyzed, that is 48 weeks post vaccination, the increase appeared to be no longer significant (S3 Table). In addition, a higher frequency of memory B cells, including class-switched memory cells, double negative cells and IgM memory cells was found in Group B as compared to Group A, although the increase was not statistically significant (S3 Fig).

### Induction of regulatory T cells correlating with levels of variable loop antibodies

Tregs were defined as CD3+ CD4+ CD25+ CD127- cells; memory cells were identified based on the expression of CCR7 and CD45RO as described by Baecher et al.[30] and Su et al. [31]; activated Tregs were defined as CD3+ CD4+ CD25+ CCR7- CD45RO+ cells, and resting Tregs cells were defined as CD3+ CD4+ CD25+ CCR7+ CD45RO+ cells (S4 Fig). While there was no difference in the frequency of Tregs between placebo and vaccinees prior to vaccination (Fig 3C), vaccination resulted in a significant induction of Tregs. Significantly higher numbers of Tregs (p<0.001) were seen at all time points post-vaccination). The median frequency of Tregs among CD4^+^ T cells was 1.2% (range 0.84–1.59%) in Group A, 2.0% (range: 0.5–2.25%) in Group B and 0.5% (range: 0.04–0.71%) in the placebo (S3 Table). Group B volunteers had a higher frequency of Tregs as compared to Group A at 1, 2 and 48 weeks post final MVA vaccination (p<0.012, p<0.009 and p<0.003 at the three time points respectively; S3 Table). We further analyzed the proportion of activated and resting Tregs among the vaccinated individuals and found that the proportion of activated Tregs was high at the time of last vaccination, but declined subsequently with time. The median frequency of activated Tregs among CD4^+^ T cells was 0.49% (range: 0.22–0.19%) in Group A, 1.125% (range 0.66–2.0%) in Group B and 0.8% (range: 0.25–1.19%) in the placebo. In contrast, the frequency of resting Tregs increased significantly within a week after final vaccination (Data not shown). We observed a positive correlation between the number of Treg cells and V1 and V3 specific antibody levels in the vaccinated individuals (p<0.035 in group A and p<0.002 in Group B respectively) (Fig 4).

## Discussion

The search for an effective HIV vaccine is still on. Some of the popular HIV vaccine trials conducted in the past including the VAX004, Merck 023 and HTN505 trials, have shown that it is difficult to elicit high titers of HIV-1-Nabs, and that the low levels of antibodies induced by the vaccines do not last long in vaccinated individuals [32]. The RV144 vaccine trial has thus far been the only HIV vaccine trial that brought some hope that a preventive HIV vaccine is possible. The trial demonstrated 31.2% reduction in risk of HIV infection among individuals vaccinated with live recombinant ALVAC-HIV(R) (vCP1521) and VaxGen gp120 B/E (AIDSVAX(R) B/E) in a prime-boost regimen [32]. A large number of immunological studies were undertaken to understand the correlates of vaccine-induced protection against HIV infection in the trial participants [33–36]. One of the interesting observations of these studies was that IgG antibodies that bind to the V1/V2 region of the HIV-1 Env correlated with lower infection rates among vaccinees [37–39]. The present study aimed to characterize the immunogenicity of the subtype C prophylactic vaccine that was tested by the Indian Council of Medical Research (ICMR) and the International AIDS Vaccine Initiative (IAVI) using MVA (TBC-M4) and ADVAX as vaccine candidates in India a few years back [6]. Preliminary analysis revealed that vaccination resulted in production of HIV-specific antibody and IFN-γ responses, and reported that though the initial response appeared to be significantly higher in the DNA prime/MVA boost group (Group A) as compared to the MVA alone group (group B), the effect lasted only for a short time, implying that both the heterologous DNA/MVA prime-boost and the homologous MVA regimens were immunologically comparable [6].

Here we describe our findings on vaccine-induced humoral immune responses studied using PBMC and plasma samples of the trial participants that were archived under ideal conditions at NIRT. The original study reported the induction of neutralizing antibodies with very limited cross-neutralization activity. Prompted by the findings of the RV144 trial, we determined the ability of the vaccine to induce V1/V2 specific antibodies, as well as antibodies to the V3 and MPER peptides of HIV-1. While none of the samples had HIV-specific antibodies binding to the variable regions or MPER peptides at baseline or immediately after first vaccination, we observed V1/V2 and V3-specific antibodies in a significant proportion of the volunteers in both the groups. On the other hand, only a small proportion of the volunteers developed MPER-specific antibody responses. Our findings are in line with previous observations that poxvirus and adenovirus-based HIV vaccines are capable of inducing antibodies to the variable regions of HIV-1 envelope. An earlier study in macaques reported a 10-fold increase in the level of anti-env antibodies following immunization with MVA alone as compared to those who were immunized with DNA and MVA [40]. A similar pattern was also observed in the trial under discussion, when total anti-env antigen specific antibody responses were measured [6]. A closely related trial called the P002 trial conducted in UK with the same vaccine construct used in the present trial, also reported higher titres of HIV-specific antibodies targeting rgp41 Env, rgp140 Env, p24 Gag, and rgp120 Env proteins in Group B than in Group A volunteers [41].

The variable loops of the HIV-1 envelope are known to be highly immunogenic in both HIV and SIV [42–43], and possess contact points for the binding of bNAbs and non-neutralising antibodies (nnAbs) [18, 19]. Multiple monoclonal bNABs targeting the V1/V2 domain (e.g. PG9, PG16, CH01, CH03, and PGT145) have been isolated from HIV-infected individuals [19, 44, 45]. However, induction of bNAbs through vaccination has remained a major challenge. While earlier studies reported that production of HIV specific bNAb correlated with clinical and viral parameters [46], most individuals with high viral loads do not develop bNAb, making it evident that the synergistic action of various factors is required for the development of bNAbs [47]. The antibodies generated in the P001 trial were found to possess very limited neutralizing activity. However, their ability to bind to gp120 peptides suggests a possible role in stimulating other protective mechanisms such as ADCC, and/or cell mediated virus inhibition [48]. Non-neutralizing epitopes of HIV expressed on the surface of infected cells may permit the destruction of these cells via ADCC, antibody-dependent cell mediated virus inhibition (ADCVI) or lysis of HIV particles via complement-mediated attack on the virus envelope [49, 50]. The lower rates of infection seen among vaccine recipients who developed HIV-1 specific nnAbs against the V2/V3 peptides in the RV144 trial provide major support for this hypothesis [38]

Tfh cells prime the B cells, support affinity maturation and play a pivotal role in antibody production [18, 19, 51–53]. Activation signals from Tfh cells are critical for the development of antibody producing B cells [54]. The frequency of Tfh cells is reported to be higher in the blood and lymphoid organs of those with AIDS as compared to HIV+ non-progressors [55]. The memory subset of Tfh cells that exist in circulation in peripheral blood are referred to as peripheral Tfh (pTfh) cells [18, 19,56–59]. Correlation between the frequency of pTfh cells in circulation and the development of broadly neutralising antibodies against HIV has been reported in a large cohort of HIV+ individuals [18]. The present study identified a significantly higher frequency of pTfh cells among vaccine recipients in both Groups A and B, as compared to the placebo. Interestingly, the frequency of pTfh cells correlated positively with levels of variable loop antibodies, suggesting that the vaccine primed the host immune system and generated pTfh cells to produce HIV-specific antibodies. Although the vaccine under study failed to elicit a potent bNAb response, it is encouraging to note that the vaccine induced Tfh cells and elicited production of variable loop specific antibodies. A positive association between Tfh cell frequency, expansion of the B cell compartment and increase in circulating high-avidity SIV-specific antibodies during chronic SIV infection have previously been reported [60], suggesting strongly that induction of Tfh cells results in a significant antibody response. However in our study, the correlation was found to be statistically significant only in Group A, although a positive correlation was also observed in Group B. The very small sample size of the present study is a major limitation in this regard.

Development of new candidate vaccines for humans against major infectious killers is known to depend largely on the quality of the antibody response. Thus, Tfh cell based vaccines have the advantage of positively influencing the generation of a robust protective immune response in humans [18,55]. Gag-specific pTfh cells are generally thought to provide help in induction of B cells, while Env-specific pTfh help in antibody class-switching [60]. Gag- and Env-specific IL-21^+^ pTfh cells upregulate the expression of cytolytic markers on HIV-specific CD8+T cells. The RV144 vaccine trial demonstrated that ALVAC+AIDSVAX induced a higher percentage of pTfh cells as compared to ALVAC alone or the DNA-Ad5 regimen like that used in the unsuccessful HVTN 505 trial. Our observations as well as that of others highlight the critical role of pTfh cells in the protective efficacy of a HIV vaccine.

Several studies have shown that the interaction between the germinal center B cells and Tfh cells through numerous surface receptors is essential for the optimal development and survival of both cell types [52, 61]. Long-term immunity to infectious agents, such as viruses, is primarily due to the expression of antibodies from plasma cells and antibody secreting cells. Studies in animal models have shown that constant replenishment of plasma cells by memory cells is not mandatory because of the existence of long-lived plasma cells [61]. However, plasma cell longevity and its role in maintaining serum antibody levels are poorly defined. In the present study, we also measured the frequency of plasma B cells and memory B cells post-vaccination and found that vaccine recipients had significantly higher numbers of plasma B cells than placebo. Group B volunteers had higher frequencies of plasma B cells than Group A vaccinees, suggesting that the homologous regimen was better in terms of plasma B cell and antibody production than the heterologous regimen. Differentiation into plasma cells and germinal center B cells greatly depends on high-affinity antigens, and Tfh cells are essential for B cell maturation, survival, and proliferation [18, 52]. Though the frequency of pTfh cells was found to be higher among the vaccinees, we could not observe any correlation with B cell frequency possibly due to the very small sample size.

Regulatory T cells (Tregs) are known to suppress activation of multiple cell types including CD4^+^ and CD8^+^ T cells, B cells, natural killer cells, and dendritic cells. In HIV infection, studies have reported that Tregs cause significant increase in antigen-specific cytokine production from HIV specific CD4+ and CD8+ T cells [62] and help to prevent reactivation of latent reservoirs of HIV [63–66]. Although there is very little data available on the induction of Tregs and their role in vaccination, Tregs are thought to play an important role in down regulating host immune response in order to maintain immune homeostasis and thus impact vaccine efficacy [67–69]. We were curious to see if vaccine under discussion had any effect on this subset of cells. Interestingly, we found that both the regimens induced Tregs without compromising the vaccine’s immunogenicity in terms of antibody production as well as induction of pTfh cells and plasma B cells. We also observed a positive correlation between the number of Treg cells and V1/V3 region antibody response in both the groups.

In summary, the present study characterized in detail the nature of the antibody response and detected V1/V2 specific antibody responses in a significant portion of volunteers, which are now known to be positive correlates for protection against HIV infection, through antibody dependent cell-mediated cytotoxicity (ADCC) or some other yet unidentified mechanism [70]. An effort to elucidate the ADCC activity of antibodies elicited by the vaccine would provide more detailed information on the protective nature of antibody response and further guidance for the design of a successful HIV-1 vaccine. Further, the variable loop antibody levels correlated positively with induction of circulating pTfh cells and Treg cells. One of the major limitations of this study is the small sample size, comprising of only 16 volunteers including 4 placebo and the remaining 12 distributed between the two study groups. Hence, many of the correlation analyses undertaken here lacked sufficient power to achieve statistical significance although positive responses were seen in many instances. However, the present study has revealed encouraging findings on the potential of the vaccine regimen for inducing favorable immunogenic responses among volunteers. Therefore, these results suggest that an in-depth analysis of vaccine-induced immune response can aid in informing future design of an HIV-1 vaccine based on the Indian Clade C virus.

## Supporting information

S1 Table Reagents used for multicolor flow cytometry. (DOCX)

S2 Table Reactivity of plasma to variable region and MPER peptides. Values represent ELISA-generated data optical density (OD) values at a wavelength of 405nM. Plasma was tested at a dilution of 1:50. Statistical analysis was performed using anova. Bonferroni post hoc test was used at 5%* level of significance.
(DOCX)

S3 Table Comparison of mean frequency of circulating T follicular helper cells, plasma B cells, regulatory T cells in placebo and vaccinees. P values were calculated using Two-way ANOVA using Bonferroni post hoc test.*—p<0.05; **—p<0.01; ***—p<0.001.
(DOCX)

S4 Table Correlation between immune cells, variable loop, and MPER-specific antibodies. (XLSX)

S1 Fig Representative pseudocolor FACS plot of circulating memory like T Follicular Helper cells. T cells were gated first on lymphocytes and then on memory T cells (CCR7+CD45RO+) followed by Tfh cells (CXCR5+PD-1+CXCR3-).
(DOCX)

S2 Fig Representative Pseudo color FACS plot of B cells and memory subsets. B cells were gated first on lymphocytes and then on plasma cells (CD38+ CD27+) and memory B cells (CD27 and IgD).
(DOCX)

S3 Fig Frequency of circulating memory B cell subsets. Graphical representation showing the % of memory B cells in placebo and vaccinees of both groups at different time points. The horizontal bars represent median and dot values represent scatter points. P values were calculated using Two-way ANOVA using Bonferroni post hoc test. */†—p<0.05; **/††—p<0.01; ***/†††—p<0.001.
(DOCX)

S4 Fig Representative pseudocolor FACS plot of regulatory T cells. T cells were gated first on lymphocytes and then on Tregs (CD4+CD127dimCD25+) followed by memory Tregs (CCR7+CD45RO+).
(DOCX)

## References

[1] UNAIDS. Prevention gap report. 2016. http://www.unaids.org/sites/default/files/mediaasset/2016-prevention-gap-report_en.pdf. Accessed 6 June 2017.

[2] Streeck H, D'souza MP, Littman DR, Crotty S. Harnessing CD4+ T cell responses in HIV vaccine development. Nature medicine. 2013;19(2):143 10.1038/nm.3054 23389614 PMC3626561

[3] Gottardo R, Bailer RT, Korber BT, Gnanakaran S, Phillips J, Shen X, et al Plasma IgG to linear epitopes in the V2 and V3 regions of HIV-1 gp120 correlate with a reduced risk of infection in the RV144 vaccine efficacy trial. PloS one. 2013;26;8(9):e75665 10.1371/journal.pone.0075665 24086607 PMC3784573

[4] Rao M, K Peachman K, Kim J, Gao G, R Alving C, L Michael N, et al HIV-1 variable loop 2 and its importance in HIV-1 infection and vaccine development. Current HIV research. 2013; 1;11(5):427–38. 24191938 10.2174/1570162x113116660064 PMC4086350

[5] Berinyuy E, Soliman ME. A broad spectrum anti-HIV inhibitor significantly disturbs V1/V2 domain rearrangements of HIV-1 gp120 and inhibits virus entry. Journal of Receptors and Signal Transduction. 2016;3;36(2):119–29. 10.3109/10799893.2015.1056307 26446906

[6] Mehendale S, Thakar M, Sahay S, Kumar M, Shete A, Sathyamurthi P, et al Safety and immunogenicity of DNA and MVA HIV-1 subtype C vaccine prime-boost regimens: a phase I randomised Trial in HIV-uninfected Indian volunteers. PLoS One. 2013;13;8(2):e55831 10.1371/journal.pone.0055831 23418465 PMC3572184

[7] Corti D, Lanzavecchia A. Broadly neutralizing antiviral antibodies. Annual review of immunology. 2013;21;31:705–42. 10.1146/annurev-immunol-032712-095916 23330954

[8] Overbaugh J, Morris L. The antibody response against HIV-1. Cold Spring Harbor perspectives in medicine. 2011;1:a007039. 10.1101/cshperspect.a007039 PMC3253031 22315717

[9] Wei X, Decker JM, Wang S, Hui H, Kappes JC, Wu X, et al Antibody neutralization and escape by HIV-1. Nature. 2003;422(6929):307 10.1038/nature01470 12646921

[10] Schiffner T, Sattentau QJ, Dorrell L. Development of prophylactic vaccines against HIV-1. Retrovirology. 2013;10(1):72. 23866844 10.1186/1742-4690-10-72 PMC3722125

[11] McCoy LE, Quigley AF, Strokappe NM, Bulmer-Thomas B, Seaman MS, Mortier D, et al Potent and broad neutralization of HIV-1 by a llama antibody elicited by immunization. Journal of Experimental Medicine. 2012; 4; 209(6):1091–103. 10.1084/jem.20112655 22641382 PMC3371729

[12] Buranapraditkun S, Pissani F, Teigler JE, Schultz BT, Alter G, Marovich M, et al Preservation of peripheral T follicular helper cell function in HIV controllers. Journal of virology. 2017; 3:JVI-00497. 10.1128/JVI.00497-17 PMC5487582 28468877

[13] Kim CH, Rott LS, Clark-Lewis I, Campbell DJ, Wu L, Butcher EC. Subspecialization of CXCR5+ T cells: B helper activity is focused in a germinal center–localized subset of CXCR5+ T cells. Journal of Experimental Medicine. 2001;18;193(12):1373–82. 11413192 10.1084/jem.193.12.1373 PMC2193300

[14] Mosmann TR, Cherwinski H, Bond MW, Giedlin MA, Coffman RL. Two types of murine helper T cell clone. I. Definition according to profiles of lymphokine activities and secreted proteins. The Journal of immunology. 1986;1;136(7):2348–57. 2419430

[15] Schaerli P, Loetscher P, Moser B. Cutting edge: induction of follicular homing precedes effector Th cell development. The Journal of Immunology. 2001;1;167(11):6082–6. 11714765 10.4049/jimmunol.167.11.6082

[16] Pallikkuth S, Pahwa S. Interleukin-21 and T follicular helper cells in HIV infection: research focus and future perspectives. Immunologic research. 2013;1;57(1–3):279–91. 10.1007/s12026-013-8457-0 24242760 PMC6510583

[17] Recher M, Berglund LJ, Avery DT, Cowan MJ, Gennery AR, Smart J, et al IL-21 is the primary common gamma chain-binding cytokine required for human B-cell differentiation in vivo. Blood. 2011;1:6824–6835. 10.1182/blood-2011-06-362533 PMC3338166 22039266

[18] Locci M, Havenar-Daughton C, Landais E, Wu J, Kroenke MA, Arlehamn CL, et al Human circulating PD-1+ CXCR3− CXCR5+ memory Tfh cells are highly functional and correlate with broadly neutralizing HIV antibody responses. Immunity. 2013;17;39(4):758–69. 10.1016/j.immuni.2013.08.031 24035365 PMC3996844

[19] Schultz BT, Teigler JE, Pissani F, Oster AF, Kranias G, Alter G, et al Circulating HIV-specific interleukin-21+ CD4+ T cells represent peripheral Tfh cells with antigen-dependent helper functions. Immunity. 2016;19;44(1):167–78. 10.1016/j.immuni.2015.12.011 26795249

[20] Mills KH, McGuirk P. Antigen-specific regulatory T cells—their induction and role in infection. InSeminars in immunology 2004;1 (Vol. 16, No. 2, pp. 107–117). 10.1016/j.smim.2003.12.006 15036234

[21] Rouse BT, Suvas S. Regulatory cells and infectious agents: detentes cordiale and contraire. The Journal of Immunology. 2004;15;173(4):2211–5. 15294929 10.4049/jimmunol.173.4.2211

[22] Mills KH. Regulatory T cells: friend or foe in immunity to infection?. Nature Reviews Immunology. 2004;4(11):841 10.1038/nri1485 15516964

[23] Johnston RJ, Poholek AC, DiToro D, Yusuf I, Eto D, Barnett B, et al Bcl6 and Blimp-1 are reciprocal and antagonistic regulators of T follicular helper cell differentiation. Science. 2009;21;325(5943):1006–10. 10.1126/science.1175870 19608860 PMC2766560

[24] Brezar V, Godot V, Cheng L, Su L, Lévy Y, Seddiki N. T-regulatory cells and vaccination “pay attention and do not neglect them”: Lessons from HIV and cancer vaccine trials. Vaccines. 2016; 5; 4(3):30. 10.3390/vaccines4030030 PMC5041024 27608046

[25] Simonetta F, Bourgeois C. CD4+ FOXP3+ regulatory T-cell subsets in human immunodeficiency virus infection. Frontiers in immunology. 2013;30;4:215 10.3389/fimmu.2013.00215 23908654 PMC3727053

[26] Chowdhury A, Del Rio PM, Tharp GK, Trible RP, Amara RR, Chahroudi A, et al Decreased T follicular regulatory cell/T follicular helper cell (TFH) in simian immunodeficiency virus–infected rhesus macaques may contribute to accumulation of TFH in chronic infection. The Journal of Immunology. 2015;21:1402701. 10.4049/jimmunol.1402701 PMC4575868 26297764

[27] O’connell RJ, Kim JH, Excler JL. The HIV-1 gp120 V1V2 loop: structure, function and importance for vaccine development. Expert review of vaccines. 2014;1;13(12):1489–500. 10.1586/14760584.2014.951335 25163695

[28] Amara RR, Villinger F, Staprans SI, Altman JD, Montefiori DC, Kozyr NL, et al Different patterns of immune responses but similar control of a simian-human immunodeficiency virus 89.6 P mucosal challenge by modified vaccinia virus Ankara (MVA) and DNA/MVA vaccines. Journal of virology. 2002;1;76(15):7625–31. 10.1128/JVI.76.15.7625-7631.2002 12097576 PMC136377

[29] Kaminski DA, Wei C, Qian Y, Rosenberg AF, Sanz I. Advances in human B cell phenotypic profiling. Frontiers in immunology. 2012;10;3:302 10.3389/fimmu.2012.00302 23087687 PMC3467643

[30] Baecher-Allan C, Viglietta V, Hafler DA. Human CD4+ CD25+ regulatory T cells. InSeminars in immunology 2004; 1 (Vol. 16, No. 2, pp. 89–98). Academic Press. 10.1016/j.smim.2003.12.005 15036232

[31] Su H, Longhi MS, Wang P, Vergani D, Ma Y. Human CD4+ CD25 high CD127 low/neg Regulatory T Cells. InHuman Cell Culture Protocols 2012 (pp. 287–299). Humana Press. 10.1007/978-1-61779-367-7_20 22057460

[32] Wang HB, Mo QH, Yang Z. HIV vaccine research: the challenge and the way forward. Journal of immunology research. 2015; 503978 10.1155/2015/503978 25861656 PMC4377490

[33] Ferrantelli F, Rasmussen RA, Hofmann-Lehmann R, Xu W, McClure HM, Ruprecht RM. Do not underestimate the power of antibodies—lessons from adoptive transfer of antibodies against HIV. Vaccine. 2002;19;20:A61–5. 12477430 10.1016/s0264-410x(02)00389-4

[34] Fauci AS, Johnston MI, Dieffenbach CW, Burton DR, Hammer SM, Hoxie JA, et al HIV vaccine research: the way forward. Science. 2008;25;321(5888):530–2. 10.1126/science.1161000 18653883

[35] Burton DR, Mascola JR. Antibody responses to envelope glycoproteins in HIV-1 infection. Nature immunology. 2015;16(6):571 10.1038/ni.3158 25988889 PMC4834917

[36] Wang Y, Sundling C, Wilson R, ODell S, Chen Y, Dai K, et al High-Resolution Longitudinal Study of HIV-1 Env Vaccine–Elicited B Cell Responses to the Virus Primary Receptor Binding Site Reveals Affinity Maturation and Clonal Persistence. The Journal of Immunology.2016; 21:1502543. 10.4049/jimmunol.1502543 PMC4868635 27001953

[37] Haynes BF, Gilbert PB, McElrath MJ, Zolla-Pazner S, Tomaras GD, Alam SM, et al Immune-correlates analysis of an HIV-1 vaccine efficacy trial. New England Journal of Medicine. 2012;5;366(14):1275–86. 10.1056/NEJMoa1113425 22475592 PMC3371689

[38] Zolla-Pazner S, Cardozo T, Karasavvas N, Gottardo R, Williams C, Morris DE, et al Analysis of V2 antibody responses induced in vaccinees in the ALVAC/AIDSVAX HIV-1 vaccine efficacy trial. PloS one. 2013;17;8(1):e53629 10.1371/journal.pone.0053629 23349725 PMC3547933

[39] Zolla-Pazner S, Gilbert PB, Williams C, Yates NL, Williams WT, Howington R, et al Vaccine-induced IgG antibodies to V1V2 regions of multiple HIV-1 subtypes correlate with decreased risk of HIV-1 infection. PloS one. 2014;4;9(2):e87572 10.1371/journal.pone.0087572 24504509 PMC3913641

[40] Barouch DH, Liu J, Li H, Maxfield LF, Abbink P, Lynch DM, et al Vaccine protection against acquisition of neutralization-resistant SIV challenges in rhesus monkeys. Nature. 2012;482(7383):89 10.1038/nature10766 22217938 PMC3271177

[41] Hayes P, Gilmour J, von Lieven A, Gill D, Clark L, Kopycinski J, et al Safety and immunogenicity of DNA prime and Modified Vaccinia Ankara virus HIV subtype C vaccine boost in healthy adults. Clinical and vaccine immunology. 2013;23:CVI–00637. 10.1128/CVI.00637-12 PMC3592345 23345581

[42] Zolla-Pazner S, Edlefsen PT, Rolland M, Kong XP, Gottardo R, Williams C, et al Vaccine-induced human antibodies specific for the third variable region of HIV-1 gp120 impose immune pressure on infecting viruses. EBioMedicine. 2014;1;1(1):37–45. 10.1016/j.ebiom.2014.10.022 25599085 PMC4293639

[43] Kent KA, Rud E, Corcoran T, Powell C, Thiriart C, Collignon C, et al Identification of two neutralizing and 8 non-neutralizing epitopes on simian immunodeficiency virus envelope using monoclonal antibodies. AIDS research and human retroviruses. 1992;8(6):1147–51. 10.1089/aid.1992.8.1147 1380261

[44] McKeating JA, Shotton C, Cordell J, Graham S, Balfe P, Sullivan N, et al Characterization of neutralizing monoclonal antibodies to linear and conformation-dependent epitopes within the first and second variable domains of human immunodeficiency virus type 1 gp120. Journal of virology. 1993;1;67(8):4932–44. 7687306 10.1128/jvi.67.8.4932-4944.1993 PMC237881

[45] Kayman SC, Wu Z, Revesz K, Chen H, Kopelman R, Pinter A. Presentation of native epitopes in the V1/V2 and V3 regions of human immunodeficiency virus type 1 gp120 by fusion glycoproteins containing isolated gp120 domains. Journal of virology. 1994;1;68(1):400–10. 7504740 10.1128/jvi.68.1.400-410.1994 PMC236300

[46] Hessell AJ, Haigwood NL. Neutralizing antibodies and control of HIV: moves and countermoves. Current HIV/AIDS Reports. 2012;1;9(1):64–72. 10.1007/s11904-011-0105-5 22203469

[47] Liao HX, Lynch R, Zhou T, Gao F, Alam SM, Boyd SD, et al Co-evolution of a broadly neutralizing HIV-1 antibody and founder virus. Nature. 2013;496(7446):469 10.1038/nature12053 23552890 PMC3637846

[48] Kramski M, Stratov I, Kent SJ. The role of HIV-specific antibody-dependent cellular cytotoxicity in HIV prevention and the influence of the HIV-1 Vpu protein. Aids. 2015; 14;29(2):137–44. 10.1097/QAD.0000000000000523 25396265

[49] Lewis GK, DeVico AL, Gallo RC. Antibody persistence and T-cell balance: two key factors confronting HIV vaccine development. Proceedings of the National Academy of Sciences. 2014; 4;111(44):15614–21. 10.1073/pnas.1413550111 PMC4226080 25349379

[50] Dörner T, Radbruch A. Antibodies and B cell memory in viral immunity. Immunity. 2007; 21;27(3):384–92. 10.1016/j.immuni.2007.09.002 17892847

[51] Kulkarni SS, Lapedes A, Tang H, Gnanakaran S, Daniels MG, Zhang M, et al Highly complex neutralization determinants on a monophyletic lineage of newly transmitted subtype C HIV-1 Env clones from India. Virology. 2009;15; 385(2):505–20. 10.1016/j.virol.2008.12.032 19167740 PMC2677301

[52] Crotty S. Follicular helper CD4 T cells (Tfh). Annual review of immunology. 2011; 23;29:621–63. 10.1146/annurev-immunol-031210-101400 21314428

[53] Ueno H. Human circulating T follicular helper cell subsets in health and disease. Journal of clinical immunology. 2016;1;36(1):34–9. 26984851 10.1007/s10875-016-0268-3

[54] Pissani F, Streeck H. Emerging concepts on T follicular helper cell dynamics in HIV infection. Trends in immunology. 2014;1;35(6):278–86. 10.1016/j.it.2014.02.010 24703588 PMC4264576

[55] Ma CS, Deenick EK, Batten M, Tangye SG. The origins, function, and regulation of T follicular helper cells. Journal of Experimental Medicine. 2012; 2;209(7):1241–53. 10.1084/jem.20120994 22753927 PMC3405510

[56] Morita R, Schmitt N, Bentebibel SE, Ranganathan R, Bourdery L, Zurawski G, et al Human blood CXCR5+ CD4+ T cells are counterparts of T follicular cells and contain specific subsets that differentially support antibody secretion. Immunity. 2011; 28;34(1):108–21. 10.1016/j.immuni.2010.12.012 21215658 PMC3046815

[57] Chevalier N, Jarrossay D, Ho E, Avery DT, Ma CS, Yu D, et al CXCR5 expressing human central memory CD4 T cells and their relevance for humoral immune responses. The Journal of Immunology. 2011;6:1002828. 10.4049/jimmunol.1002828 21471443

[58] He J, Tsai LM, Leong YA, Hu X, Ma CS, Chevalier N, et al Circulating precursor CCR7loPD-1hi CXCR5+ CD4+ T cells indicate Tfh cell activity and promote antibody responses upon antigen reexposure. Immunity. 2013;17; 39(4):770–81. 10.1016/j.immuni.2013.09.007 24138884

[59] Bentebibel SE, Lopez S, Obermoser G, Schmitt N, Mueller C, Harrod C, et al Induction of ICOS+ CXCR3+ CXCR5+ TH cells correlates with antibody responses to influenza vaccination. Science translational medicine. 2013;13;5(176):176ra32 10.1126/scitranslmed.3005191 23486778 PMC3621097

[60] Petrovas C, Yamamoto T, Gerner MY, Boswell KL, Wloka K, Smith EC, et al CD4 T follicular helper cell dynamics during SIV infection. The Journal of clinical investigation. 2012; 4;122(9):3281–94. 10.1172/JCI63039 22922258 PMC3428091

[61] Hammarlund E, Thomas A, Amanna IJ, Holden LA, Slayden OD, Park B, et al Plasma cell survival in the absence of B cell memory. Nature communications. 2017;24;8(1):1781 10.1038/s41467-017-01901-w 29176567 PMC5701209

[62] Aandahl EM, Michaëlsson J, Moretto WJ, Hecht FM, Nixon DF. Human CD4+ CD25+ regulatory T cells control T-cell responses to human immunodeficiency virus and cytomegalovirus antigens. Journal of virology. 2004;1;78(5):2454–9. 10.1128/JVI.78.5.2454-2459.2004 14963140 PMC369239

[63] Chase AJ, Yang HC, Zhang H, Blankson JN, Siliciano RF. Preservation of FoxP3+ regulatory T cells in the peripheral blood of human immunodeficiency virus type 1-infected elite suppressors correlates with low CD4+ T-cell activation. Journal of virology. 2008; 1;82(17):8307–15. 10.1128/JVI.00520-08 18579608 PMC2519624

[64] Kared H, Lelièvre JD, Donkova-Petrini V, Aouba A, Melica G, Balbo M, et al HIV-specific regulatory T cells are associated with higher CD4 cell counts in primary infection. AIDS (London, England). 2008;22(18):2451. 10.1097/QAD.0b013e328319edc0 PMC3195674 19005268

[65] Simonetta F, Bourgeois C. CD4+ FOXP3+ regulatory T-cell subsets in human immunodeficiency virus infection. Frontiers in immunology. 2013;30;4:215 10.3389/fimmu.2013.00215 23908654 PMC3727053

[66] Li G, Nunoya JI, Reszka-Blanco N, Cheng L, Tsao LC, Su L. Efficient activation of latent HIV reservoir in human memory T cells by depletion of regulatory T cells in humanized mice in vivo. J Immunol,2017;1:198 (1 Supplement) 78.21.

[67] Brezar V, Ruffin N, Richert L, Surenaud M, Lacabaratz C, Palucka K, et al Decreased HIV-specific T-regulatory responses are associated with effective DC-vaccine induced immunity. PLoS pathogens. 2015;27;11(3):e1004752 10.1371/journal.ppat.1004752 25816350 PMC4376642

[68] Boer MC, Joosten SA, Ottenhoff TH. Regulatory T-cells at the interface between human host and pathogens in infectious diseases and vaccination. Frontiers in immunology. 2015; 11;6:217 10.3389/fimmu.2015.00217 26029205 PMC4426762

[69] Ndure J, Flanagan KL. Targeting regulatory T cells to improve vaccine immunogenicity in early life. Frontiers in microbiology. 2014;11;5:477 10.3389/fmicb.2014.00477 25309517 PMC4161046

[70] Kramski M, Stratov I, Kent SJ. The role of HIV-specific antibody-dependent cellular cytotoxicity in HIV prevention and the influence of the HIV-1 Vpu protein. AIDS. 2015; 14;29(2):137–44. 10.1097/QAD.0000000000000523 25396265

