# Correction: Induction of circulating T follicular helper cells and regulatory T cells correlating with HIV-1 gp120 variable loop antibodies by a subtype C prophylactic vaccine tested in a Phase I trial in India

**DOI:** 10.1371/journal.pone.0204476

**Published:** 2018-09-19

**Authors:** The PLOS ONE Staff

## Notice of Republication

This article was republished on September 7, 2018 to correct an error to the ORCID iD of the corresponding author introduced during the typesetting process. The publisher apologizes for the error. Please download this article again to view the correct version.

In addition, the corresponding author’s name has been corrected. The correct name is: Hanna Elizabeth Luke.
