# Supplementary material for: Induction of circulating T follicular helper cells and regulatory T cells correlating with HIV-1 gp120 variable loop antibodies by a subtype C prophylactic vaccine tested in a Phase I trial in India
Source: PLoS One. 2018 Aug 29;13(8):e0203037. doi: 10.1371/journal.pone.0203037 (PMC6114930; doi:10.1371/journal.pone.0203037)
Supplement: S1 Table — (DOCX) [file pone.0203037.s001.docx]

**S1Table.** **Commercial reagents used for multicolor flow cytometry**

Panel for T follicular Helper cells:

| **Specificity** | **Flurochrome** | **Ab Clone** | **Vendor** | **Catalog Number** | **Vol/test** |
| --- | --- | --- | --- | --- | --- |
| CD3 | APC H7 | SK7 | BD-Pharminogen | 557832 | 2.5ul |
| CD4 | BUV737 | RPA-T4 | BD-Pharminogen | 564305 | 5ul |
| CD45 RO | BUV395 | UCHL1 | BD-Pharminogen | 564291 | 5ul |
| CCR7 | PeCY7 | G043H7 | BD-Pharminogen | 557648 | 5ul |
| CXCR3 | APC R 700 | IC6 | BD-Pharminogen | 561320 | 5ul |
| CXCR5 | BB515 | RF8B2 | BD-Pharminogen | 564624 | 5ul |
| PD-1 | PE | EH12.1 | BD-Pharminogen | 560795 | 15ul |
| Dead cells | Aqua Blue | -- | Invitrogen | L34957 | 1:20 |

Panel for B cells:

| **Specificity** | **Flurochrome** | **Ab Clone** | **Vendor** | **Catalog Number** | **Vol/test** |
| --- | --- | --- | --- | --- | --- |
| CD3 | APC H7 | SK7 | BD-Pharminogen | 557832 | 2.5ul |
| CD38 | APC | HIT2 | BD-Pharminogen | 555462 | 5ul |
| CD19 | BUV395 | 2H7 | BD-Pharminogen | 563519 | 5ul |
| CD20 | PE | HIB19 | BD-Pharminogen | 555623 | 5ul |
| IgD | BUV737 | IA6-2 | BD-Pharminogen | 564687 | 5ul |
| CD27 | BB515 | O323 | BD-Pharminogen | 564642 | 5ul |
| Dead cells | Aqua Blue | -- | Invitrogen | L34957 | 1:20 |

Panel for Treg cells:

| **Specificity** | **Flurochrome** | **Ab Clone** | **Vendor** | **Catalog Number** | **Vol/test** |
| --- | --- | --- | --- | --- | --- |
| CD3 | APC H7 | SK7 | BD-Pharminogen | 557832 | 2.5ul |
| CD4 | BUV737 | RPA-T4 | BD-Pharminogen | 564305 | 5ul |
| CD45 RO | BUV395 | UCHL1 | BD-Pharminogen | 564291 | 5ul |
| CCR7 | PeCY7 | G043H7 | BD-Pharminogen | 557648 | 5ul |
| CD127 | PE-CF594 | HIL-7R-M21 | BD-Pharminogen | 562397 | 5ul |
| CD25 | APC | M-A251 | BD-Pharminogen | 555434 | 5ul |
| Dead cells | Aqua Blue | -- | Invitrogen | L34957 | 1:20 |

**S2 Table. Reactivity of plasma to variable region and MPER peptides** Values represent ELISA-generated data optical density (OD) values at a wavelength of 405nM. Plasma was tested at a dilution of 1:50. Statistical analysis was performed using anova. Bonferroni post hoc test was used at 5%* level of significance.

| **Table: Elisa OD Values at wavelength of 405 nM** | | | | | |
| --- | --- | --- | --- | --- | --- |
| **Antigen** | **Day** | **Group** | | | **Sig.** |
|  |  | **Placebo (n = 4)** | **A (n = 6)** | **B (n = 6)** |  |
| **MPER** | **Baseline** | 0.115 (0.092 - 0.144) | 0.101 (0.099 - 0.134) | 0.148 (0.117 - 0.163) | **0.257** |
|  | **I^st^ wk post Vac.2** | 0.129 (0.101 - 0.147) | 0.109 (0.107 - 0.129) | 0.144 (0.118 - 0.169) | **0.584** |
|  | **Vac .3** | 0.151 (0.117 - 0.195) | 0.202 (0.143 - 0.234) | 0.139 (0.124 - 0.173) | **0.369** |
|  | **2^st^ wks post Vac.3** | 0.126 (0.101 - 0.161) | 0.131 (0.117 - 0.164) | 0.144 (0.112 - 0.173) | **0.831** |
| **V1** | **Baseline** | 0.138 (0.108 - 0.177) | 0.135 (0.118 - 0.206) | 0.166 (0.118 - 0.219) | **0.570** |
|  | **I^st^ wk post Vac.2** | 0.134 (0.099 - 0.174) | 0.151 (0.116 - 0.192) | 0.151 (0.113 - 0.175) | **0.682** |
|  | **Vac .3** | 0.199 (0.140 - 0.284) | 0.225 (0.221 - 0.522) | 0.176 (0.151 - 0.194) | **0.199** |
|  | **2^st^ wks post Vac.3** | 0.200 (0.138 - 0.261) | 0.160 (0.153 - 0.450) | 0.189 (0.148 - 0.324) | **0.718** |
| **V2** | **Baseline** | 0.121 (0.097 - 0.141) | 0.122 (0.099 - 0.149) | 0.119 (0.115 - 0.135) | **0.883** |
|  | **I^st^ wk post Vac.2** | 0.121 (0.099 - 0.150) | 0.133 (0.117 - 0.150) | 0.118 (0.104 - 0.123) | **0.416** |
|  | **Vac .3** | 0.174 (0.132 - 0.241) | 0.242 (0.156 - 0.384) | 0.138 (0.114 - 0.164) | **0.210** |
|  | **2^st^ wks post Vac.3** | 0.171 (0.141 - 0.208) | 0.192 (0.168 - 0.296) | 0.150 (0.125 - 0.167) | **0.160** |
| **V3** | **Baseline** | 0.152 (0.112 - 0.164) | 0.121 (0.103 - 0.161) | 0.137 (0.108 - 0.179) | **0.944** |
|  | **I^st^ wk post Vac.2** | 0.177 (0.126 - 0.185) | 0.119 (0.109 - 0.161) | 0.149 (0.120 - 0.174) | **0.701** |
|  | **Vac .3** | 0.184 (0.133 - 0.281) | 0.360 (0.191 - 0.440) | 0.206 (0.157 - 0.231) | **0.203** |
|  | **2^st^ wks post Vac.3** | 0.215 (0.166 - 0.224) | 0.399 (0.355 - 1.146) | 0.553 (0.232 - 0.958) | **0.041** |

**S3 Table. Comparison of mean frequency of circulating T follicular helper cells, plasma B cells, regulatory T cells in placebo and vaccinees.** P values were calculated using Two-way ANOVA using Bonferroni post hoc test.* - p<0.05; ** - p<0.01; *** - p<0.001.

|  | | | | | | | | | | | | |
| --- | --- | --- | --- | --- | --- | --- | --- | --- | --- | --- | --- | --- |
| **Visit** | **B cells** | | | **P Value** | **Treg** | | | **P Value** | **Tfh** | | | **P Value** |
|  | **Placebo** | **Group A** | **Group B** |  | **Placebo** | **Group A** | **Group B** |  | **Placebo** | **Group A** | **Group B** |  |
| 0-V | 0.53  (0.41 - 0.72) | 0.99  (0.74 - 1.42) | 0.93  (0.74 - 1.07) | 0.118 | 0.33  (0.30 - 0.39) | 0.40  0.31 - 0.44) | 0.59  (0.52 - 0.69) | 0.027  (P vs. B*) | 0.10  (0.04 - 0.27) | 0.08  (0.04 - 0.08) | 0.18  (0.08 - 0.30) | 0.005  (P vs. B*;  A vs. B **) |
| 1^st^ MVA | 0.78  (0.60 - 0.96) | 2.10  (1.02 - 2.62) | 1.29  (0.98 - 1.42) | 0.026  (P vs. A*) | 0.40  (0.36 - 0.44) | 0.85  (0.79 - 0.86) | 0.72  (0.62 - 0.80) | 0.008  (P vs. A**) | 0.22  (0.18 – 0.26) | 0.20  (0.13 - 0.37) | 0.34  (0.23 - 0.47) | 0.010  (P vs. A*;  P vs. B**) |
| 1 Week Post 1^st^ MVA | 0.79  (0.70 - 0.94) | 2.12  (1.92 - 2.40) | 2.33  (1.78 - 2.64) | 0.014  (P vs. A*;  P vs. B**) | 0.66  (0.51 - 0.85) | 1.18  (0.74 - 1.38) | 1.16  (1.10 - 1.44) | 0.061 | 0.09  (0.05 – 0.10) | 0.14  (0.11 - 0.17) | 0.15  (0.05 - 0.17) | 0.004  (P vs. B**;  A vs. B*) |
| Last MVA | 0.52  (0.45 - 0.60) | 2.14  (1.32 - 2.21) | 2.23  (2.03 - 2.36) | 0.013  (P vs. A*;  P vs. B**) | 0.56  (0.41 - 0.66) | 1.50  (1.21 - 1.79) | 1.26  (1.05 - 1.55) | 0.013  (P vs. A**;  P vs. B*) | 0.10  (0.07 - 0.14) | 0.15  (0.07 - 0.20) | 0.18  (0.10 - 0.15) | 0.013  (P vs. A**;  P vs. B**) |
| 1 Week Post last  MVA | 0.79  (0.66 - 0.87) | 2.13  (2.01 - 2.15) | 3.01  (2.08 - 3.64) | 0.010  (P vs. A*;  P vs. B**) | 0.67  (0.58 - 0.73) | 1.70  (1.41 - 2.14) | 2.04  (1.48 - 2.28) | 0.012  (P vs. A*;  P vs. B**) | 0.12  (0.05 - 0.34) | 0.16  (0.07 - 0.31) | 0.09  (0.06 - 0.09) | 0.006  (P vs. B**) |
| 2 Week Post  Last MVA | 0.37  (0.18 - 0.58) | 1.23  (0.74 - 1.61) | 2.79  (2.46 - 4.31) | 0.004  (P vs. B**) | 0.65  (0.58 - 0.74) | 1.50  (1.4 0- 1.59) | 2.34  (1.40 - 2.59) | 0.009  (P vs. A*;  P vs. B**) | 0.05  (0.03 - 0.07) | 0.06  (0.04 - 0.12) | 0.10  (0.07 - 0.13) | 0.005  (P vs. B**) |
| 48 Week Post  Last MVA | 0.47  (0.26 - 0.55) | 1.29  (0.91 - 1.76) | 1.43  (1.37 - 1.47) | 0.086 | 0.76  (0.71 - 0.9) | 1.61  (1.25 - 1.9) | 2.68 (2.34 - 2.92) | 0.003  (P vs. B***) | 0.03  (0.02 - 0.07) | 0.05  (0.02 - 0.11) | 0.07  (0.05 - 0.08) | 0.016  (P vs. A*; P vs. B**) |
| Diff. bet. a Grp - Sig. | < 0.001 (P vs. A***; P vs. B***) | | | | < 0.001 (P vs. A***; P vs. B***) | | | | < 0.001 (P vs. A***; P vs. B***; A vs. B***) | | | |
| Values were presented as Median (inter quartile range).  P - Placebo; A - Group A; B - Group B. * < 0.05, ** < 0.01, *** < 0.001 | | | | | | | | | | | | |
|  | | |  |  |  |  |  |  |  |  |  |  |

**S1Fig.Representative pseudocolor FACS plot of circulating memory like T Follicular Helper cells.**

T cells were gated first on lymphocytes and then on memory T cells (CCR7+CD45RO+) followed by Tfh cells (CXCR5+PD-1+CXCR3-).

**
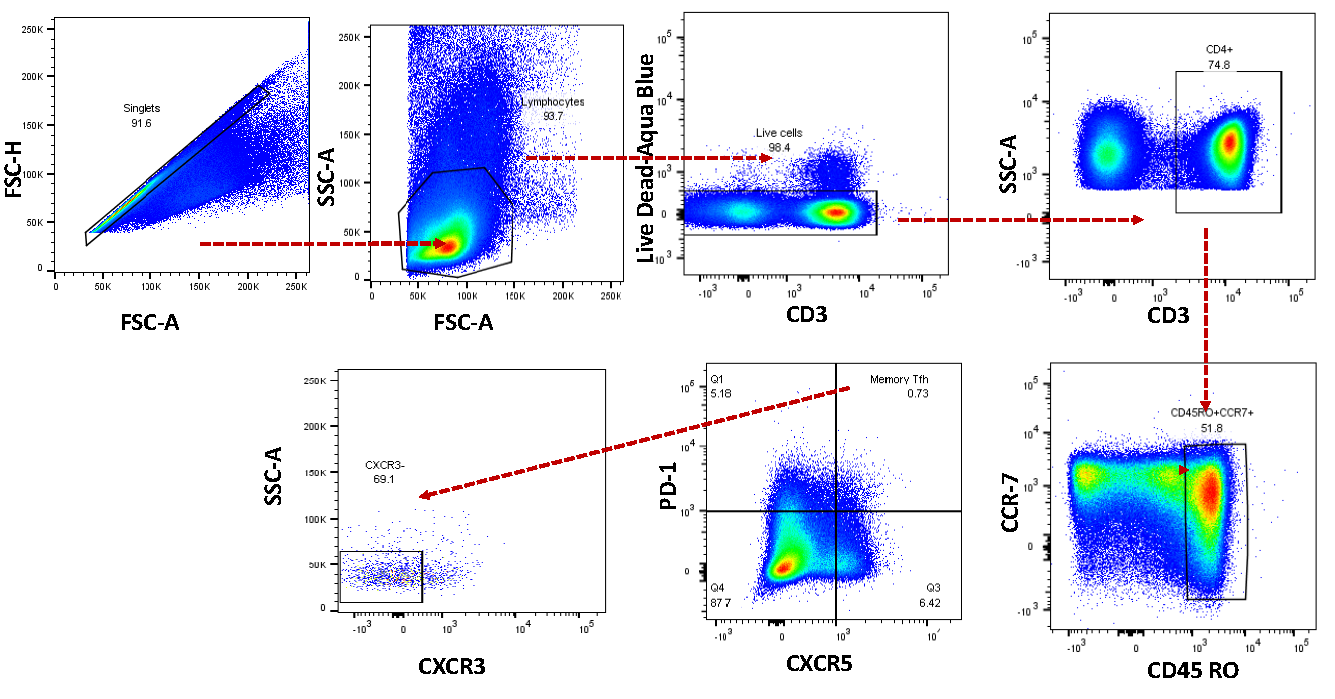
**

**S2 Fig. Representative Pseudo color FACS plot of B cells and memory subsets**.

B cells were gated first on lymphocytes and then on plasma cells (CD38+ CD27+) and memory B cells (CD27 and IgD).

**
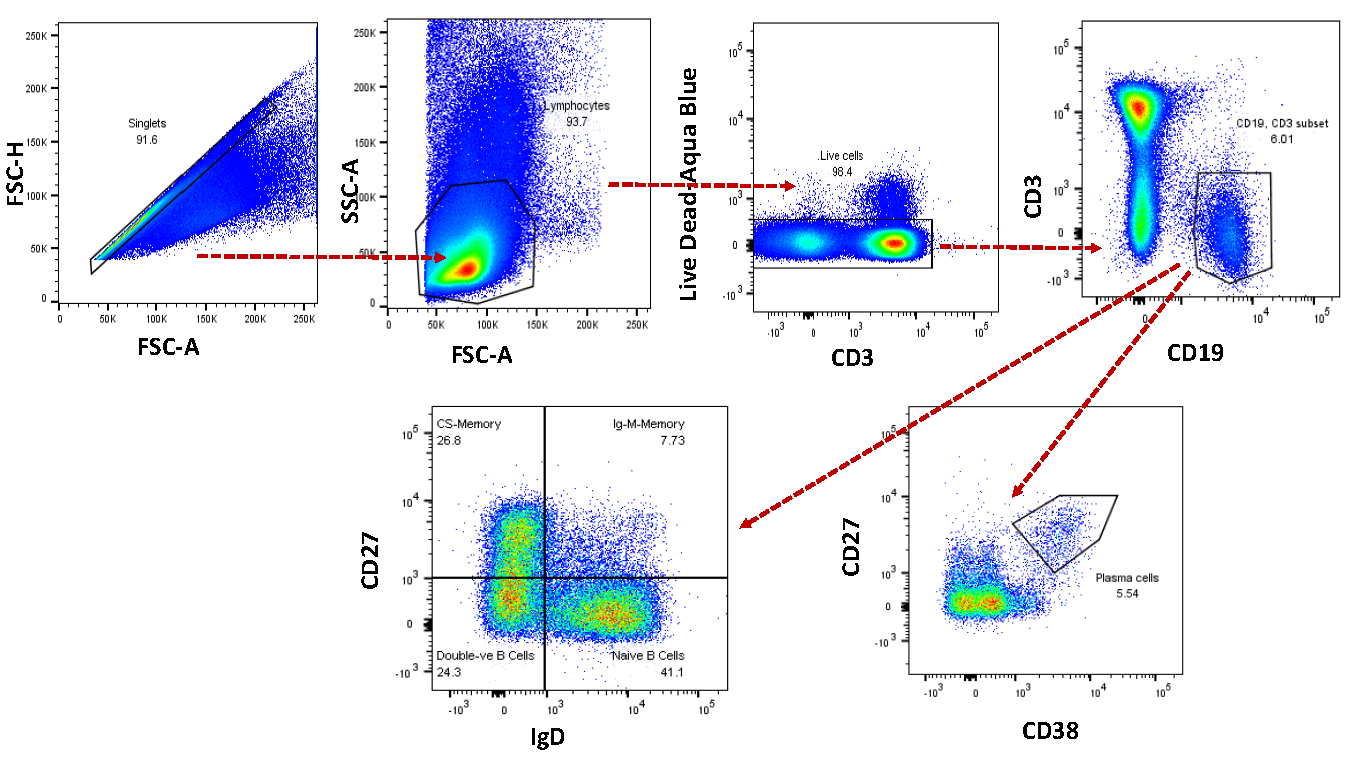
**

**S3 Fig. Frequency of circulating memory B cell subsets.** Graphical representation showing the % of memory B cells in placebo and vaccinees of both groups at different time points. The horizontal bars represent median and dot values represent scatter points. P values were calculated using Two-way ANOVA using Bonferroni post hoc test. */† - p<0.05; **/†† - p<0.01; ***/††† - p<0.001


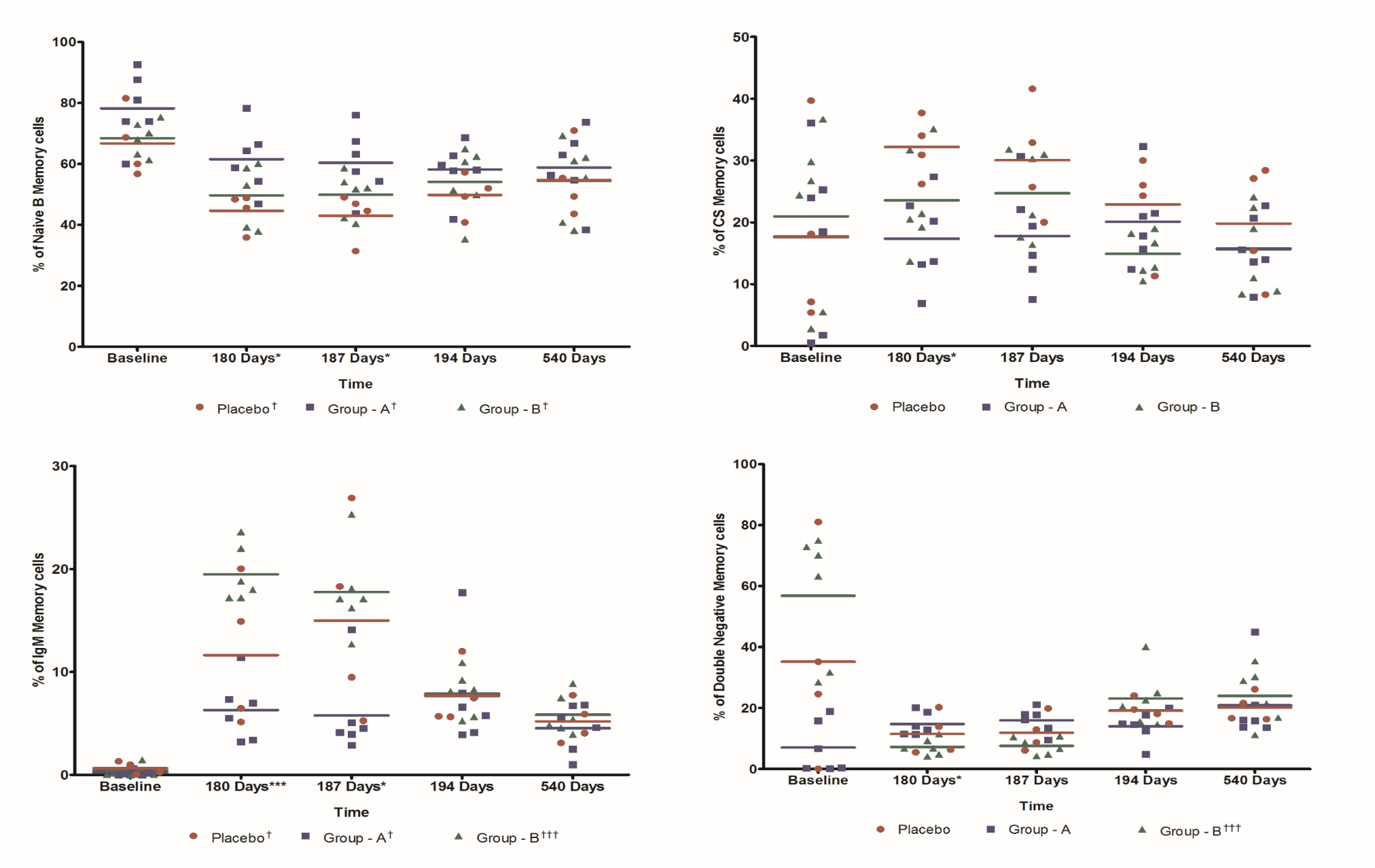


**S4 Fig. Representative pseudocolor FACS plot of regulatory T cells.**

T cells were gated first on lymphocytes and then on Tregs (CD4+CD127dimCD25+) followed by memory Tregs (CCR7+CD45RO+).

**
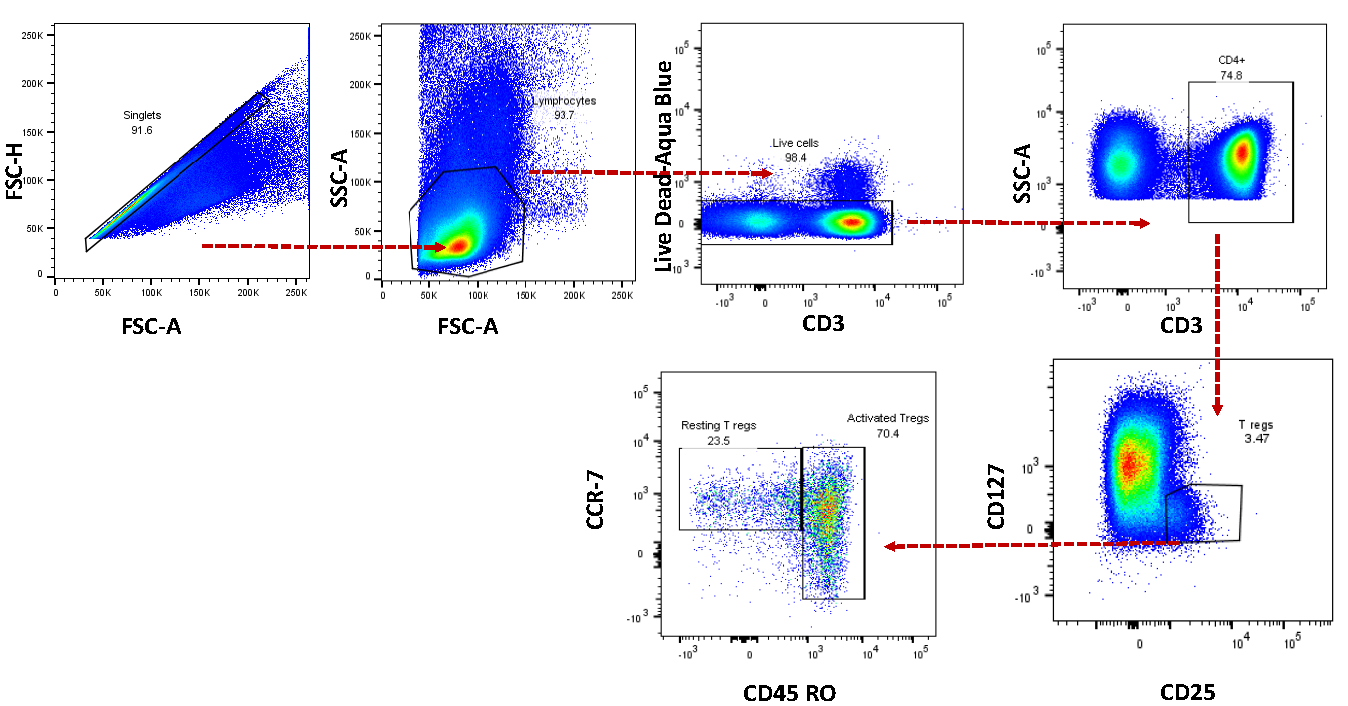
**
