# Supplementary material for: Induction of circulating T follicular helper cells and regulatory T cells correlating with HIV-1 gp120 variable loop antibodies by a subtype C prophylactic vaccine tested in a Phase I trial in India
Source: PLoS One. 2018 Aug 29;13(8):e0203037. doi: 10.1371/journal.pone.0203037 (PMC6114930; doi:10.1371/journal.pone.0203037)
Supplement: S2 Table — Values represent ELISA-generated data optical density (OD) values at a wavelength of 405nM. Plasma was tested at a dilution of 1:50. Statistical analysis was performed using anova. Bonferroni post hoc test was used at 5%* level of significance. (DOCX) [file pone.0203037.s002.docx]

**S2 Table. Reactivity of plasma to variable region and MPER peptides** Values represent ELISA-generated data optical density (OD) values at a wavelength of 405nM. Plasma was tested at a dilution of 1:50. Statistical analysis was performed using anova. Bonferroni post hoc test was used at 5%* level of significance.

| **Table: Elisa OD Values at wavelength of 405 nM** | | | | | |
| --- | --- | --- | --- | --- | --- |
| **Antigen** | **Day** | **Group** | | | **Sig.** |
|  |  | **Placebo (n = 4)** | **A (n = 6)** | **B (n = 6)** |  |
| **MPER** | **Baseline** | 0.115 (0.092 - 0.144) | 0.101 (0.099 - 0.134) | 0.148 (0.117 - 0.163) | **0.257** |
|  | **I^st^ wk post Vac.2** | 0.129 (0.101 - 0.147) | 0.109 (0.107 - 0.129) | 0.144 (0.118 - 0.169) | **0.584** |
|  | **Vac .3** | 0.151 (0.117 - 0.195) | 0.202 (0.143 - 0.234) | 0.139 (0.124 - 0.173) | **0.369** |
|  | **2^st^ wks post Vac.3** | 0.126 (0.101 - 0.161) | 0.131 (0.117 - 0.164) | 0.144 (0.112 - 0.173) | **0.831** |
| **V1** | **Baseline** | 0.138 (0.108 - 0.177) | 0.135 (0.118 - 0.206) | 0.166 (0.118 - 0.219) | **0.570** |
|  | **I^st^ wk post Vac.2** | 0.134 (0.099 - 0.174) | 0.151 (0.116 - 0.192) | 0.151 (0.113 - 0.175) | **0.682** |
|  | **Vac .3** | 0.199 (0.140 - 0.284) | 0.225 (0.221 - 0.522) | 0.176 (0.151 - 0.194) | **0.199** |
|  | **2^st^ wks post Vac.3** | 0.200 (0.138 - 0.261) | 0.160 (0.153 - 0.450) | 0.189 (0.148 - 0.324) | **0.718** |
| **V2** | **Baseline** | 0.121 (0.097 - 0.141) | 0.122 (0.099 - 0.149) | 0.119 (0.115 - 0.135) | **0.883** |
|  | **I^st^ wk post Vac.2** | 0.121 (0.099 - 0.150) | 0.133 (0.117 - 0.150) | 0.118 (0.104 - 0.123) | **0.416** |
|  | **Vac .3** | 0.174 (0.132 - 0.241) | 0.242 (0.156 - 0.384) | 0.138 (0.114 - 0.164) | **0.210** |
|  | **2^st^ wks post Vac.3** | 0.171 (0.141 - 0.208) | 0.192 (0.168 - 0.296) | 0.150 (0.125 - 0.167) | **0.160** |
| **V3** | **Baseline** | 0.152 (0.112 - 0.164) | 0.121 (0.103 - 0.161) | 0.137 (0.108 - 0.179) | **0.944** |
|  | **I^st^ wk post Vac.2** | 0.177 (0.126 - 0.185) | 0.119 (0.109 - 0.161) | 0.149 (0.120 - 0.174) | **0.701** |
|  | **Vac .3** | 0.184 (0.133 - 0.281) | 0.360 (0.191 - 0.440) | 0.206 (0.157 - 0.231) | **0.203** |
|  | **2^st^ wks post Vac.3** | 0.215 (0.166 - 0.224) | 0.399 (0.355 - 1.146) | 0.553 (0.232 - 0.958) | **0.041** |
