# Supplementary material for: Induction of circulating T follicular helper cells and regulatory T cells correlating with HIV-1 gp120 variable loop antibodies by a subtype C prophylactic vaccine tested in a Phase I trial in India
Source: PLoS One. 2018 Aug 29;13(8):e0203037. doi: 10.1371/journal.pone.0203037 (PMC6114930; doi:10.1371/journal.pone.0203037)
Supplement: S3 Table — P values were calculated using Two-way ANOVA using Bonferroni post hoc test.*—p<0.05; **—p<0.01; ***—p<0.001. (DOCX) [file pone.0203037.s003.docx]

**S3 Table. Comparison of mean frequency of circulating T follicular helper cells, plasma B cells, regulatory T cells in placebo and vaccinees.** P values were calculated using Two-way ANOVA using Bonferroni post hoc test.* - p<0.05; ** - p<0.01; *** - p<0.001.

|  | | | | | | | | | | | | |
| --- | --- | --- | --- | --- | --- | --- | --- | --- | --- | --- | --- | --- |
| **Visit** | **B cells** | | | **P Value** | **Treg** | | | **P Value** | **Tfh** | | | **P Value** |
|  | **Placebo** | **Group A** | **Group B** |  | **Placebo** | **Group A** | **Group B** |  | **Placebo** | **Group A** | **Group B** |  |
| 0-V | 0.53  (0.41 - 0.72) | 0.99  (0.74 - 1.42) | 0.93  (0.74 - 1.07) | 0.118 | 0.33  (0.30 - 0.39) | 0.40  0.31 - 0.44) | 0.59  (0.52 - 0.69) | 0.027  (P vs. B*) | 0.10  (0.04 - 0.27) | 0.08  (0.04 - 0.08) | 0.18  (0.08 - 0.30) | 0.005  (P vs. B*;  A vs. B **) |
| 1^st^ MVA | 0.78  (0.60 - 0.96) | 2.10  (1.02 - 2.62) | 1.29  (0.98 - 1.42) | 0.026  (P vs. A*) | 0.40  (0.36 - 0.44) | 0.85  (0.79 - 0.86) | 0.72  (0.62 - 0.80) | 0.008  (P vs. A**) | 0.22  (0.18 – 0.26) | 0.20  (0.13 - 0.37) | 0.34  (0.23 - 0.47) | 0.010  (P vs. A*;  P vs. B**) |
| 1 Week Post 1^st^ MVA | 0.79  (0.70 - 0.94) | 2.12  (1.92 - 2.40) | 2.33  (1.78 - 2.64) | 0.014  (P vs. A*;  P vs. B**) | 0.66  (0.51 - 0.85) | 1.18  (0.74 - 1.38) | 1.16  (1.10 - 1.44) | 0.061 | 0.09  (0.05 – 0.10) | 0.14  (0.11 - 0.17) | 0.15  (0.05 - 0.17) | 0.004  (P vs. B**;  A vs. B*) |
| Last MVA | 0.52  (0.45 - 0.60) | 2.14  (1.32 - 2.21) | 2.23  (2.03 - 2.36) | 0.013  (P vs. A*;  P vs. B**) | 0.56  (0.41 - 0.66) | 1.50  (1.21 - 1.79) | 1.26  (1.05 - 1.55) | 0.013  (P vs. A**;  P vs. B*) | 0.10  (0.07 - 0.14) | 0.15  (0.07 - 0.20) | 0.18  (0.10 - 0.15) | 0.013  (P vs. A**;  P vs. B**) |
| 1 Week Post last  MVA | 0.79  (0.66 - 0.87) | 2.13  (2.01 - 2.15) | 3.01  (2.08 - 3.64) | 0.010  (P vs. A*;  P vs. B**) | 0.67  (0.58 - 0.73) | 1.70  (1.41 - 2.14) | 2.04  (1.48 - 2.28) | 0.012  (P vs. A*;  P vs. B**) | 0.12  (0.05 - 0.34) | 0.16  (0.07 - 0.31) | 0.09  (0.06 - 0.09) | 0.006  (P vs. B**) |
| 2 Week Post  Last MVA | 0.37  (0.18 - 0.58) | 1.23  (0.74 - 1.61) | 2.79  (2.46 - 4.31) | 0.004  (P vs. B**) | 0.65  (0.58 - 0.74) | 1.50  (1.4 0- 1.59) | 2.34  (1.40 - 2.59) | 0.009  (P vs. A*;  P vs. B**) | 0.05  (0.03 - 0.07) | 0.06  (0.04 - 0.12) | 0.10  (0.07 - 0.13) | 0.005  (P vs. B**) |
| 48 Week Post  Last MVA | 0.47  (0.26 - 0.55) | 1.29  (0.91 - 1.76) | 1.43  (1.37 - 1.47) | 0.086 | 0.76  (0.71 - 0.9) | 1.61  (1.25 - 1.9) | 2.68 (2.34 - 2.92) | 0.003  (P vs. B***) | 0.03  (0.02 - 0.07) | 0.05  (0.02 - 0.11) | 0.07  (0.05 - 0.08) | 0.016  (P vs. A*; P vs. B**) |
| Diff. bet. a Grp - Sig. | < 0.001 (P vs. A***; P vs. B***) | | | | < 0.001 (P vs. A***; P vs. B***) | | | | < 0.001 (P vs. A***; P vs. B***; A vs. B***) | | | |
| Values were presented as Median (inter quartile range).  P - Placebo; A - Group A; B - Group B. * < 0.05, ** < 0.01, *** < 0.001 | | | | | | | | | | | | |
