# Supplementary material for: Induction of circulating T follicular helper cells and regulatory T cells correlating with HIV-1 gp120 variable loop antibodies by a subtype C prophylactic vaccine tested in a Phase I trial in India
Source: PLoS One. 2018 Aug 29;13(8):e0203037. doi: 10.1371/journal.pone.0203037 (PMC6114930; doi:10.1371/journal.pone.0203037)
Supplement: S1 Fig — T cells were gated first on lymphocytes and then on memory T cells (CCR7+CD45RO+) followed by Tfh cells (CXCR5+PD-1+CXCR3-). (DOCX) [file pone.0203037.s005.docx]

**S1Fig.Representative pseudocolor FACS plot of circulating memory like T Follicular Helper cells.**

T cells were gated first on lymphocytes and then on memory T cells (CCR7+CD45RO+) followed by Tfh cells (CXCR5+PD-1+CXCR3-).

**
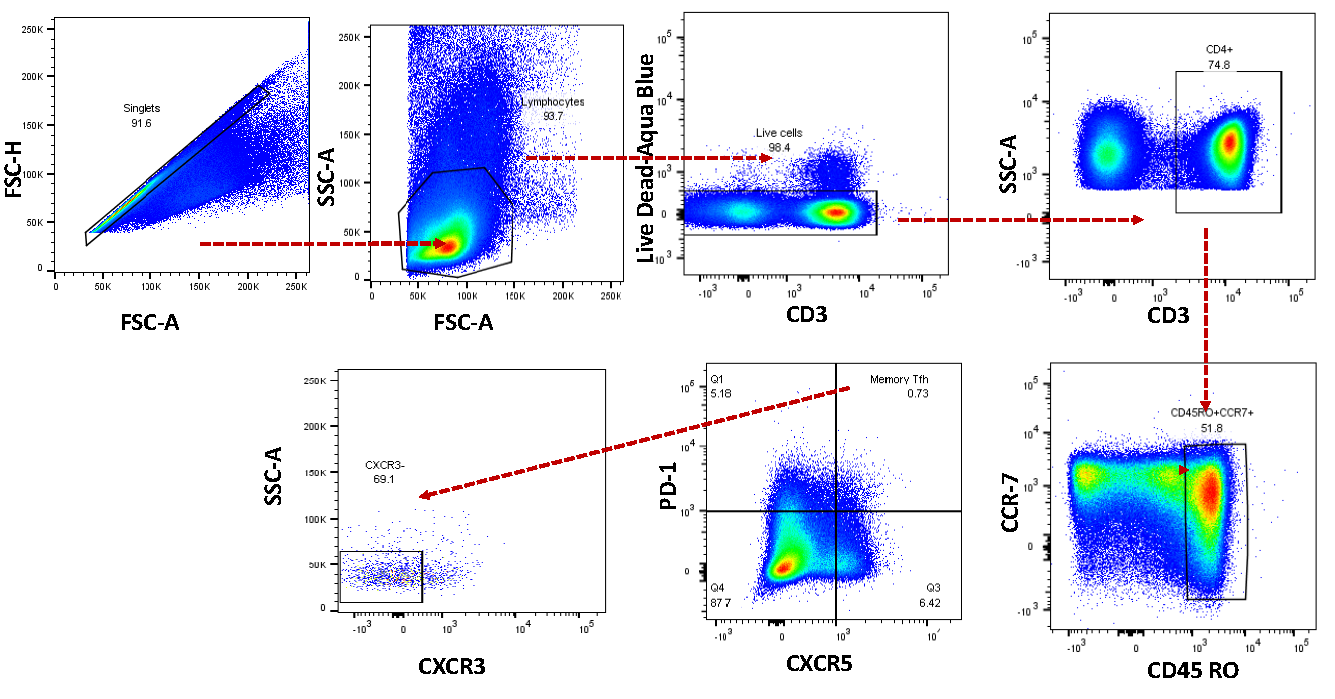
**
