# Supplementary material for: Induction of circulating T follicular helper cells and regulatory T cells correlating with HIV-1 gp120 variable loop antibodies by a subtype C prophylactic vaccine tested in a Phase I trial in India
Source: PLoS One. 2018 Aug 29;13(8):e0203037. doi: 10.1371/journal.pone.0203037 (PMC6114930; doi:10.1371/journal.pone.0203037)
Supplement: S2 Fig — B cells were gated first on lymphocytes and then on plasma cells (CD38+ CD27+) and memory B cells (CD27 and IgD). (DOCX) [file pone.0203037.s006.docx]

**S2 Fig. Representative Pseudo color FACS plot of B cells and memory subsets**.

B cells were gated first on lymphocytes and then on plasma cells (CD38+ CD27+) and memory B cells (CD27 and IgD).

**
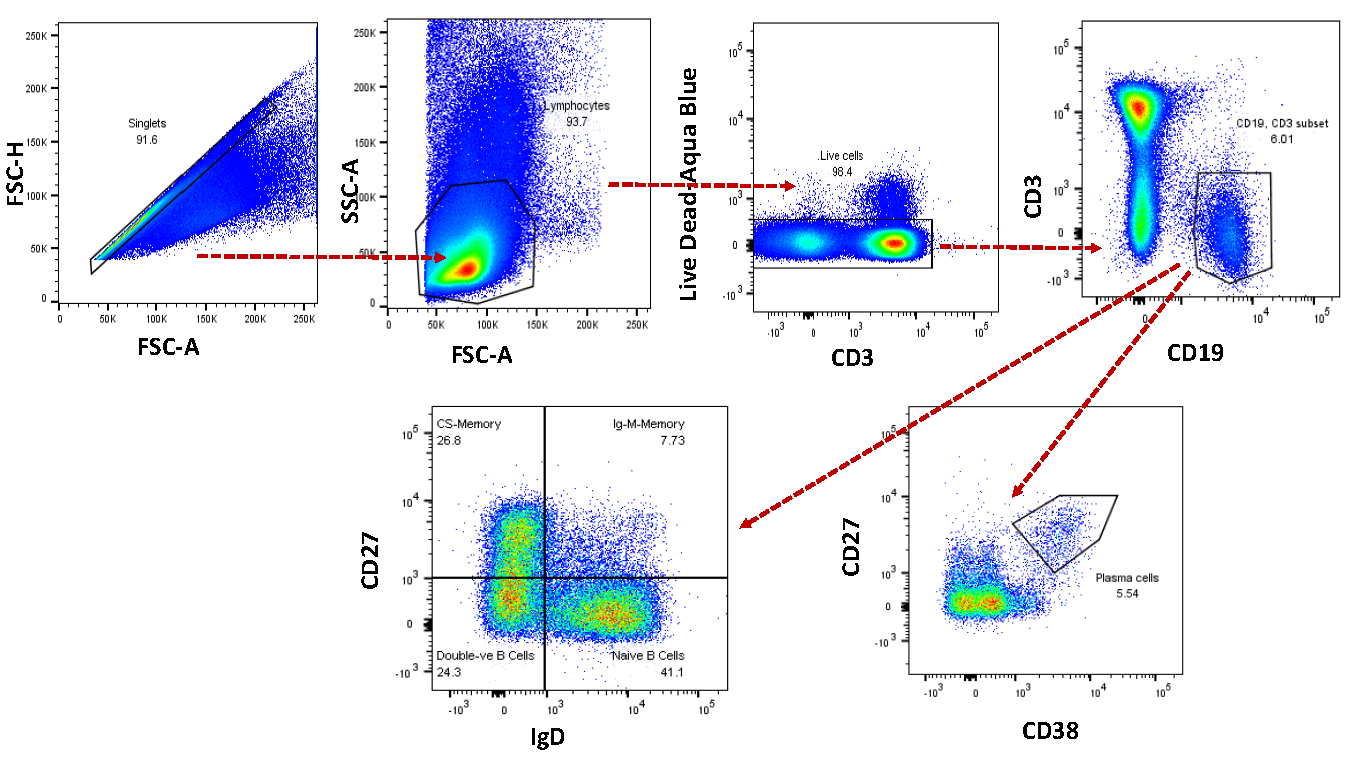
**
