# Supplementary material for: Induction of circulating T follicular helper cells and regulatory T cells correlating with HIV-1 gp120 variable loop antibodies by a subtype C prophylactic vaccine tested in a Phase I trial in India
Source: PLoS One. 2018 Aug 29;13(8):e0203037. doi: 10.1371/journal.pone.0203037 (PMC6114930; doi:10.1371/journal.pone.0203037)

**S3 Fig. Frequency of circulating memory B cell subsets.** Graphical representation showing the % of memory B cells in placebo and vaccinees of both groups at different time points. The horizontal bars represent median and dot values represent scatter points. P values were calculated using Two-way ANOVA using Bonferroni post hoc test. */† - p<0.05; **/†† - p<0.01; ***/††† - p<0.001


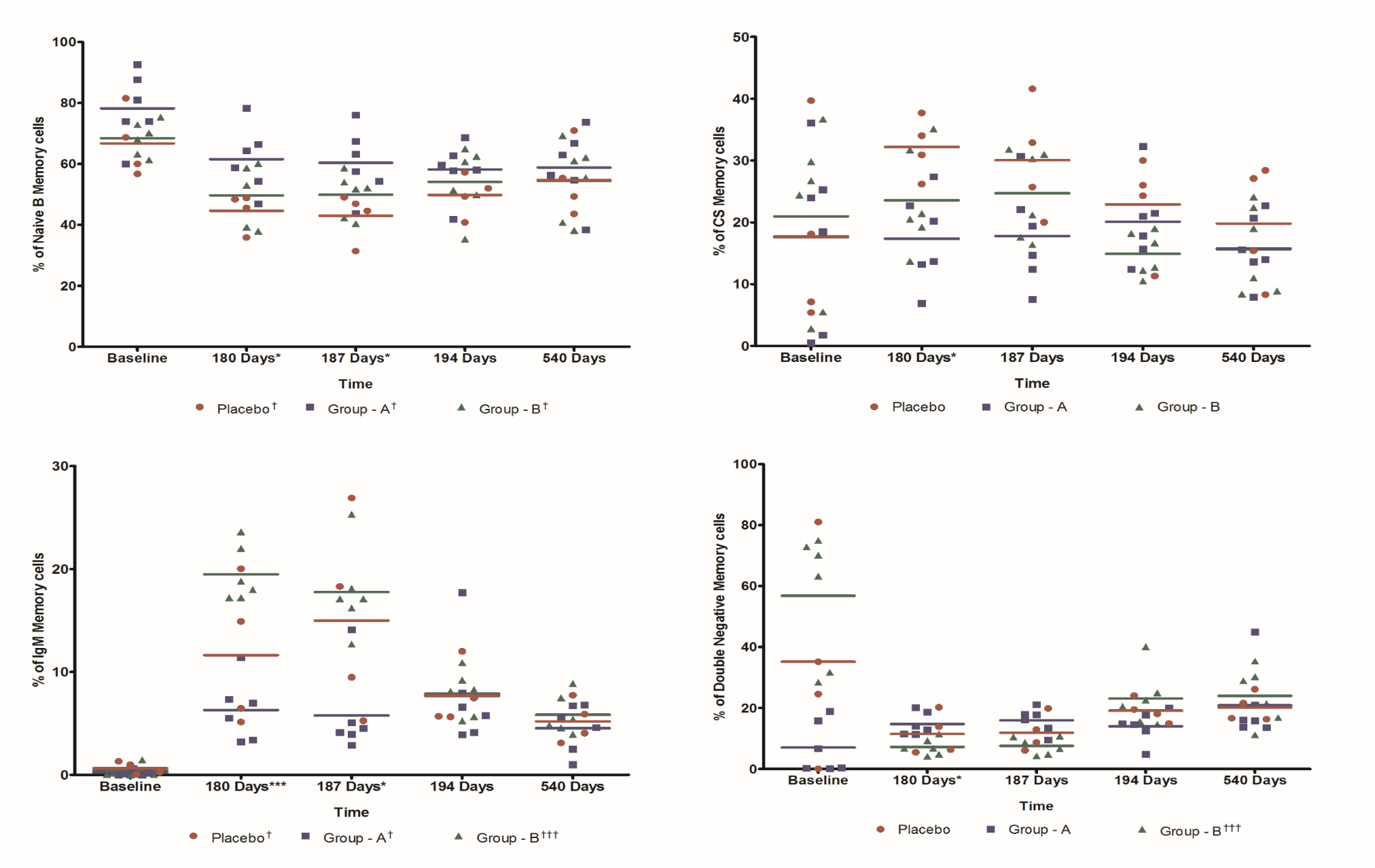

Supplement: S3 Fig — Graphical representation showing the % of memory B cells in placebo and vaccinees of both groups at different time points. The horizontal bars represent median and dot values represent scatter points. P values were calculated using Two-way ANOVA using Bonferroni post hoc test. */†—p<0.05; **/††—p<0.01; ***/†††—p<0.001. (DOCX) [file pone.0203037.s007.docx]
