# Supplementary material for: Induction of circulating T follicular helper cells and regulatory T cells correlating with HIV-1 gp120 variable loop antibodies by a subtype C prophylactic vaccine tested in a Phase I trial in India
Source: PLoS One. 2018 Aug 29;13(8):e0203037. doi: 10.1371/journal.pone.0203037 (PMC6114930; doi:10.1371/journal.pone.0203037)
Supplement: S4 Fig — T cells were gated first on lymphocytes and then on Tregs (CD4+CD127dimCD25+) followed by memory Tregs (CCR7+CD45RO+). (DOCX) [file pone.0203037.s008.docx]

**S4 Fig. Representative pseudocolor FACS plot of regulatory T cells.**

T cells were gated first on lymphocytes and then on Tregs (CD4+CD127dimCD25+) followed by memory Tregs (CCR7+CD45RO+).

**
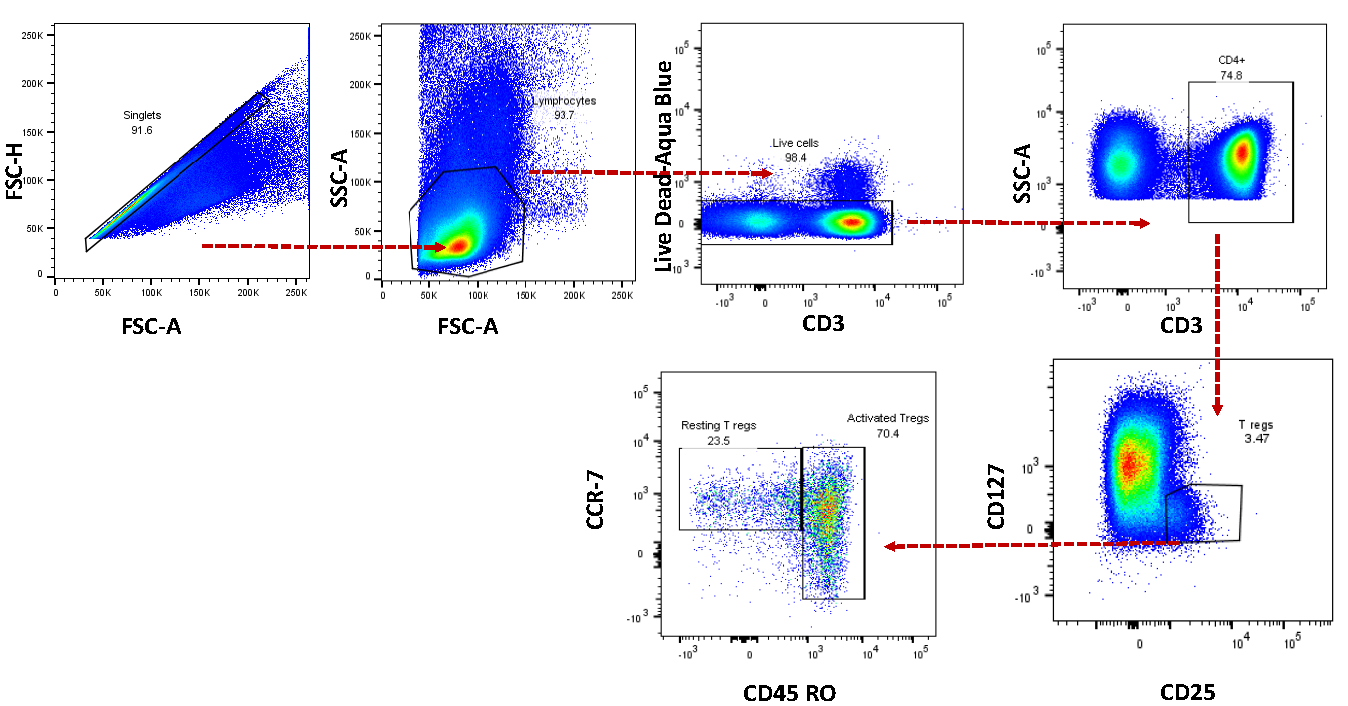
**
